# Bubble wall confinement–driven molecular assembly toward sub–12 nm and beyond precision patterning

**DOI:** 10.1126/sciadv.adf3567

**Published:** 2023-03-15

**Authors:** Zhiyuan Qu, Peng Zhou, Fanyi Min, Shengnan Chen, Mengmeng Guo, Zhandong Huang, Shiyang Ji, Yongli Yan, Xiaodong Yin, Hanqiu Jiang, Yubin Ke, Yong Sheng Zhao, Xuehai Yan, Yali Qiao, Yanlin Song

**Affiliations:** ^1^ Beijing National Laboratory for Molecular Sciences, Key Laboratory of Green Printing, CAS Research, Institute of Chemistry, Chinese Academy of Sciences, Beijing 100190, P. R. China.; ^2^ University of Chinese Academy of Sciences, Beijing 100049, P. R. China.; ^3^ State Key Laboratory of Biochemical Engineering, Institute of Process Engineering, Chinese Academy of Sciences, Beijing 100190, P. R. China.; ^4^ School of Chemical Engineering and Technology, Xi'an JiaoTong University, Shaanxi 710049, P. R. China.; ^5^ Key Laboratory of Photochemistry, Institute of Chemistry, Chinese Academy of Sciences, Beijing 100190, P. R. China.; ^6^ Key Laboratory of Cluster Science, Ministry of Education of China, Beijing Key Laboratory of Photoelectronic/Electrophotonic Conversion Materials, School of Chemistry and Chemical Engineering, Beijing Institute of Technology, Beijing 102488, P. R. China.; ^7^ Institute of High Energy Physics, Chinese Academy of Sciences, Beijing 100049, P. R. China.; ^8^ Spallation Neutron Source Science Center, Dongguan 523803, P. R. China.

## Abstract

Patterning is attractive for nanofabrication, electron devices, and bioengineering. However, achieving the molecular-scale patterns to meet the demands of these fields is challenging. Here, we propose a bubble-template molecular printing concept by introducing the ultrathin liquid film of bubble walls to confine the self-assembly of molecules and achieve ultrahigh-precision assembly up to 12 nanometers corresponding to the critical point toward the Newton black film limit. The disjoining pressure describing the intermolecular interaction could predict the highest precision effectively. The symmetric molecules exhibit better reconfiguration capacity and smaller preaggregates than the asymmetric ones, which are helpful in stabilizing the drainage of foam films and construct high-precision patterns. Our results confirm the robustness of the bubble template to prepare molecular-scale patterns, verify the criticality of molecular symmetry to obtain the ultimate precision, and predict the application potential of high-precision organic patterns in hierarchical self-assembly and high-sensitivity sensors.

## Teaser

An efficient bubble-template printing method is developed for fabrication of nano- and molecular-scale patterns.

## INTRODUCTION

Patterning holds great promise for application in nanofabrication (*1*) and nanomaterials assembly (*2*, *3*), with demonstrated prospects in the fabrication of nano/molecular-scale devices (*4*). Printing ultrahigh-precision patterns at the molecular scale are extremely attractive for the high-sensitivity sensors (*5*), photoelectric functional devices (*4*, *6*), and tissue engineering (*7*, *8*). The incompatibility of traditional photolithography technology with organic functional molecules limits its application (*6*). In the past few decades, many nonlithographic patterning methods have been developed (*9*). Among them, ultrahigh-precision patterning methods for organic molecules mainly include dip-pen nanolithography (DPN) (*10*–*12*), nanoimprint lithography (NI-L)(*13*–*15*), block copolymer self-assembly (BCPA) (*16*–*18*), and DNA origami lithography (DNAo-L) (*19*, *20*). DPN technology involves the molecular transfer in the meniscus and chemisorption on the surface; thus, the factors affecting the ultimate resolution are complex and difficult to control. Nanoimprinting is a kind of replication technology, and it is difficult to precisely manipulate the assembly behavior of molecules. BCPA uses the microphase separation of polymers and thus is incapable for direct patterning organic small molecules. DNAo-L uses the molecular characteristics of base-pair complementation, while molecular generalization is quite limited. Therefore, an efficient approach to achieving ultraprecise molecular self-assembly is the key to constructing molecular scale patterns.

The Langmuir-Blodgett technique can realize two-dimensional (2D) molecular-scale ultrathin films with a controllable number of layers at the gas-liquid interface (*21*), while there are still huge challenges in patterning molecules. In nature, the foam system is composed of bubbles with a large specific surface area, and the thickness of the liquid film between the bubbles can reach tens of nanometers or even two layers of molecules (Newton black film, ca. a few nanometers for most amphipathic functional molecules) (*22*), which has great potential for preparing ultrahigh-precision patterns. Theoretically, the ultimate thickness of the bubble wall can reach the Newton black film of bilayer thickness, which provides an ideal platform for ultrahigh-precision 1D molecular patterning.

Here, we propose a bubble-template molecular printing (BTMP) approach by introducing the ultrathin liquid film of bubble walls as a soft confinement space. Using a two-fragment molecular modal system, patterns with width up to sub–12 nm are achieved, which consisted of six layers of molecules. We point out that disjoining pressure isotherm curve could be used to predict the highest pattern precision for various model molecules. The aggregation-induced emission (AIE) π-conjugated core structure capably enables the in situ visualization of the bubble evolution process, especially the bubble rupture stage, which is notable for understanding the diversity and uniformity of pattern morphologies. Furthermore, we experimentally and theoretically reveal the molecular assembly mechanism in the bubble wall confinement space and demonstrate that the symmetric molecules exhibit much better reconfiguration capacity and smaller preaggregates, which are key points to stabilize the foam film drainage and construct higher-precision bulk phase–dominant patterns. It could be expected that asymmetric structures are more favorable to reaching the ultimate bilayer molecular precision due to the denser film at the gas-liquid interface. These findings provide a powerful solution for 1D molecular assembly, as well as ultrahigh-precision patterning and multiple-functionalized application.

## RESULTS

### Concept and process for BTMP

**Fig. 1.**
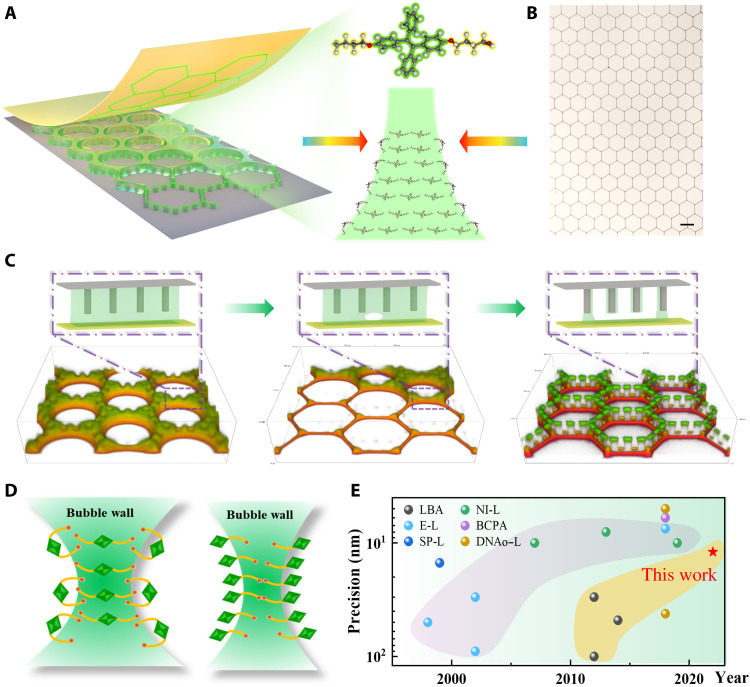
BTMP method. (**A**) Schematic diagram of BTMP, including bubble template, functional molecules, and printing patterns. (**B**) Optical image of *TPE-diSDS* molecular patterns. Scale bar, 100 μm. (**C**) Three main steps in BTMP revealed by an in situ confocal microscope, including uniform bubble array formation, bubble wall rupture, and liquid bridge shrinkage. (**D**) The forecast of pattern precision limit for symmetric and asymmetric molecules. (**E**) The precision comparison of BTMP (this work) with other methods for organic materials, including edge lithography (E-L), liquid bridge self-assembly (LBA), scanning probe lithography (SP-L), nanoimprint lithography (NI-L), block copolymer self-assembly (BCPA) and DNA origami lithography (DNAo-L). Pink highlighted areas represent the patterning methods based on traditional photolithography technique, and the yellow areas represent the patterning methods based on liquid bridge.

BTMP is a convenient approach to printing molecular patterns. First, the rational design of the template with columnar microstructure can arbitrarily tune the curvature of the gas-liquid interface, and the resulting anti-Oswald gas transport leads to well-controlled bubble arrays with diverse topologies (*23*, *24*), including triangle, square, rhombus, hexagon, and other complex tessellation patterns (fig. S1). Second, the structure of organic functional molecules is critical when these bubble arrays are used as the template to induce molecular assembly (*25*). We bring out a two-fragment strategy, including both functional and surface-active units that are connected through alkyl chains. In particular, *TPE-diSDS* (fig. S2) is chosen, which retains the surface activity characteristic from the sodium sulfonate group (surface tension of 53 mN/m; fig. S3A) to ensure the stability of bubbles while inheriting the AIE on-state property from the tetraphenylethene group (5 to 30 mg/ml; fig. S3B) to facilitate the in situ visualization. Last, the operation of molecular printing is simply conducted with a sandwich structure on the arbitrary targeting substrate (rigid or flexible substrate; fig. S4), which makes bubble template a universal and scalable strategy for molecular patterns (Fig. 1, A and B). Figure 1C shows the three stages in the BTMP process visualized in situ by the confocal microscope, including uniform bubble array formation, bubble wall rupture, and liquid bridge shrinkage. Initially, the columnar microstructure on the template is evenly wrapped by *TPE-diSDS* solution, and the bubbles present a quasi-2D morphology. The time-dependent fluorescence intensity curve (fig. S5) can accurately distinguish this stage from the latter two because the molecular concentration remains unchanged during the gas transport process at a time scale of 6 to 8 min (*26*). With the occurrence of drainage and drying process, the bubble wall becomes thinner and thinner until the rupture occurs. At this critical point, the gas in the bubble is connected to the atmosphere, and the morphology of the bubble rapidly evolves from the ellipsoid of the quasi-2D bubble to the hexagonal liquid bridge distributed along with the template, as shown in movie S1. With the gradual expansion of the rupture area, a skeleton structure around both the template and substrate is formed, leading to the molecular patterns on the substrate. Pattern precision achieved by BTMP method, up to 12 nm composed of six ordered molecular layers, proves that it is a promising molecular-level patterning technique and comparable to most state-of-the-art molecular patterning methods (Fig. 1E and table S1), including the patterning method based on traditional photolithography technique.

### Morphological control and bubble rupture position

**Fig. 2.**
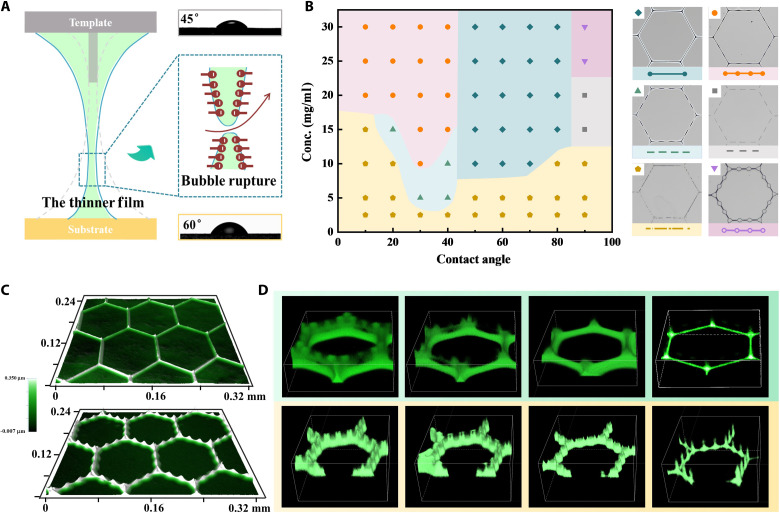
Morphological control of BTMP. (**A**) Schematic diagram of bubble rupture location control based on various wettability of substrate and template. Bubble wall with solid line represents that the thinner foam film forms near the substrate and tends to rupture when the CA of the template is smaller than the substrate (45° and 60°). The dotted line represents the opposite. (**B**) Phase diagram of the various molecular patterns versus the CA of substrate and the concentration of molecules. The CA of template is kept at 45° in this map. The molecular patterns assembled by BTMP method includes six-line morphologies, uniform line (pale-green diamond), inhomogeneous line (orange sphere), broken line 1 (green upward triangle), broken line 2 (gray square), dotted line (brown pentagon), and bead line (purple downward triangle). (**C**) Surface profiler results of uniform line and inhomogeneous line. (**D**) In situ visualization of different morphology evolution revealed by the 2D reconstruction of the fluorescence imaging results.

The in situ observation of bubble evolution is important to control molecular assembly; in particular, the manipulation of rupture position is the key to achieving molecular patterns with uniform and diverse morphologies (Fig. 2A). As shown in the assembly phase diagram, six kinds of pattern morphologies are realized (Fig. 2B). Specifically, the solution concentration is a key factor affecting the molecular assembly morphology. Relative low concentration could cause the molecules to become less tightly packed and result in a noncontinuous line morphology. The critical lowest concentration can be estimated from the mean molecular area of the dense membrane at the gas-liquid interface in the pi-A isotherm curve (3 mg/ml; fig. S6B). On the other hand, the relative magnitude of the contact angles (CA) of the substrate and template is the determining factor to adjust the spatial location of the bubble rupture and, thus, the morphology uniformity. When the CA of the substrate is smaller than that of the template, the bubble wall in the silicon column side is thinner and tends to rupture, leading to an intact liquid bridge below the silicon column and then the inhomogeneous line morphology (Fig. 2C). The rupture position can be well controlled to occur between the top of silicon column and the substrate, leading to ruptured liquid bridge and thus achieving the uniform line morphology. All these results have been verified by 3D reconstruction of the fluorescence images for the whole evolution process (Fig. 2D). The scanning electron microscopy (SEM) results of silicon columns with different printing conditions also confirm this process (fig. S7).

### Precision control of BTMP

**Fig. 3.**
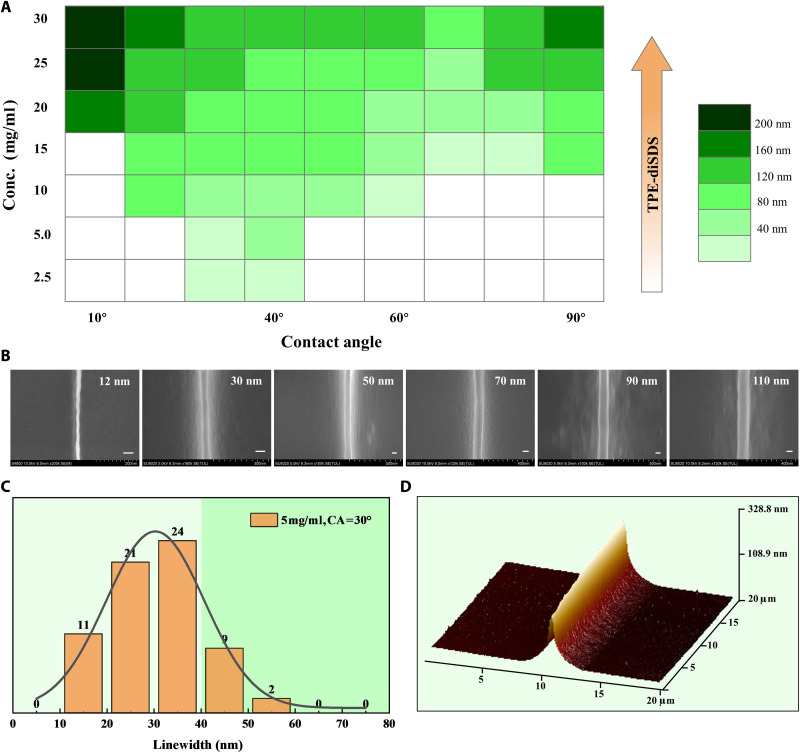
Precision control of BTMP. (**A**) Phase diagram of various linewidth versus CA of the substrate and the concentration of solutions. The CA of template is kept at 10° in this map to maximize the room to get the uniform line. (**B**) SEM images of molecular patterns with different feature sizes from 12 to 110 nm. Scale bars, 50 nm. (**C**) Distribution histograms of the linewidth in a special experiment condition, CA = 30° and concentration = 5.0 mg/ml. (**D**) The lateral morphology of a line in the patterns revealed by AFM.

On the basis of the two-fragment molecular strategy, the BTMP method realizes ultrahigh-precision patterns at the molecular level. Through statistical analysis of the molecular pattern widths under different printing conditions, controllable precision can be achieved from ten to several hundred nanometers (Fig. 3, A and B, and fig. S8, A to C), which exhibits a great correlation with the initial concentration of the solution (fig. S9A) and the CA of the substrate. Taking the results of CA = 30° and 5 mg/ml (Fig. 3C and fig. S9B) as an example, the linewidth in most areas is well controlled within the range of 20 to 40 nm. The atomic force microscopy (AFM) result (Fig. 3D) shows that the high-precision molecular pattern exhibits a sharp peak morphology, and transmission electron microscopy (TEM) images (fig. S8, D and E) further exhibit an extremely thin molecular layer at the bottom, which is attributed to the natural shrinking process of the bubble wall at the gas-liquid and liquid-solid interfaces, respectively. To verify the robustness of the BTMP method, another model molecular *TPE-SDS* with asymmetric hydrophilic-hydrophobic structure has been designed and synthesized (figs. S3C and S6, A and B). The phase diagram of *TPE-SDS* (fig. S10) indicates that it follows similar regulation rules for precision control to that of *TPE-diSDS*. However, the highest precision can only reach 50 nm, much lower than that of *TPE-diSDS*.

### Regulation mechanism: The dynamic evolution of foam film and molecular aggregation

**Fig. 4.**
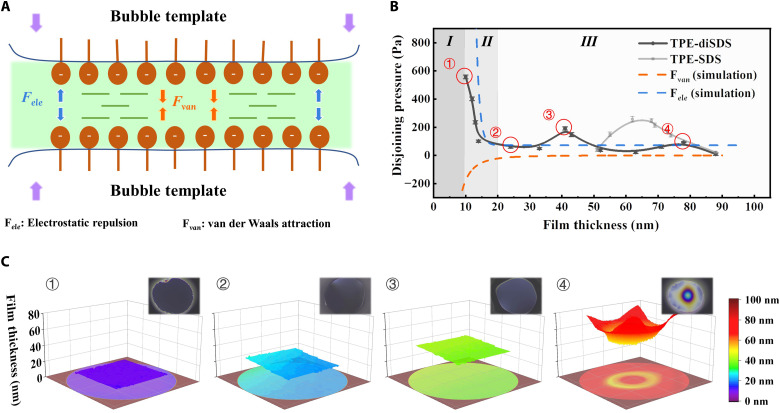
The ultrathin film evolution in BTMP method. (**A**) Schematic diagram of bubble wall thinning mechanism, which is driven by capillary force and pressure on macro-level, electrostatic repulsion and van der Waals attraction on the molecular level. (**B**) Disjoining pressure isothermal curve, electrostatic force, and van der Waals force of model molecules. The positive value means repulsion, and the negative value means attraction. Various phases I, II, and III in evolution represent Newton black film, common black film, and thin film individually. (**C**) Thin film morphology and corresponding thickness maps revealed by in situ optical microscope and the interferometry digital imaging optical microscopy protocols. The serial number corresponds to (B).

The regulation mechanism of pattern precision in BTMP method is systematically investigated from two aspects: One is concerned with the soft confinement space, like the dynamic evolution of thin liquid film in the foam system (Fig. 4A), and the other is related to the functional molecules, like the dynamic evolution of molecular aggregation during the confined assembly process. As for the former aspect, a disjoining pressure isothermal curve, including electrostatic double-layer force, London–van der Waals force, and other forces arising from supramolecular structure, can help predict the ultimate thickness of bubble walls. Accordingly, a Scheludko-like cell (*22*) combined with the reflected light microscopy (*27*) is constructed to measure the disjoining pressure and film thickness synchronously. The closed cell composed of a plane-parallel film and surrounding meniscus is used to imitate a single bubble wall and its plateau border (*28*). The drainage from the film into the meniscus occurs spontaneously by boosting the container pressure. As for *TPE-SDS*, the foam film drains rapidly and finally ruptures around 50 nm (Fig. 4B and fig. S11). This unstable foam film drainage process is decisive to the final thickness of bubble walls, which is well consistent with the corresponding pattern precision of 40 to 50 nm. As for *TPE-diSDS*, we find that foam films can go through a continuous and steady drainage (Fig. 4, B and C, and fig. S12) up to around 10 nm, beyond which there will be an obvious repulsive force appearing in disjoining pressure isothermal curve and preventing the bubble wall from further shrinking. The electrostatic double-layer repulsion of interfacial molecular layers should be the greatest resistance. In BTMP method, it is still challenging to push the shrinkage of bubble wall to bilayer ultimate precision, which highly relies on the elaborate design of molecular architectures with appropriate intermolecular attractive and repulsive interactions (*22*, *28*). Nevertheless, it is worth noting that the symmetric *TPE-diSDS* model system has already realized 12-nm precision, which lies in the range of common black film (ca. 10 to 20 nm for most amphipathic functional molecules) in foam physics (*22*). The result implies great potential of the BTMP method to approach the ultimate Newton black film thickness through the effective combination of delicate molecular choice and precise regulation of disjoining pressure.

**Fig. 5.**
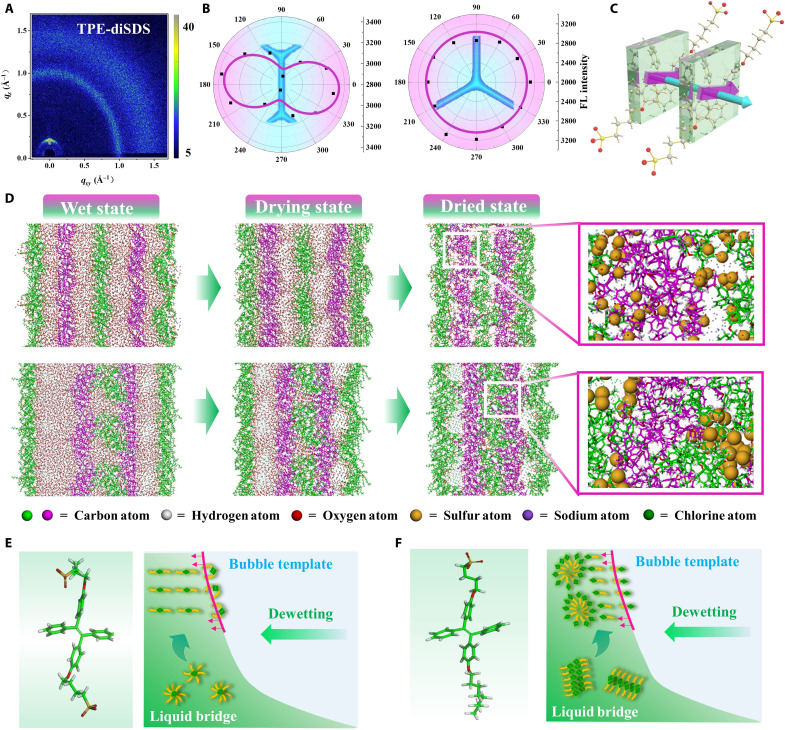
Molecular assembly mechanism of BTMP. (**A**) GIWAXS result of *TPE-diSDS* solid patterns. (**B**) The fluorescence microscopy images and spatial polarization profile of light emission for the solid patterns in line and in intersection. The former exhibits fluorescence anisotropy at an angle of 70°, and the latter exhibits isotropy. (**C**) Schematic diagram of the relationship between the molecular transition dipole moment (purple), the molecular stacking mode (green), and the direction of patterns (blue). (**D**) MD simulation results of *TPE-diSDS* pattern (top) and *TPE-SDS* pattern (bottom) in wet state, drying state, and solid state. There are three components in the simulation system, including printed molecules (*TPE-diSDS* or *TPE-SDS*), water molecules, and other inorganic ions. The magnification boxes show the configuration of *TPE-diSDS* and *TPE-SDS* in solid patterns. The size of sulfur atoms has been enlarged to distinguish molecular configuration easily. (**E**) Schematic diagram of *TPE-diSDS* aggregation and stacking molds. These assemble to spherical micelle in solution and transfer to well-organized out-of-plane layered structure after BTMP. (**F**) Schematic diagram of *TPE-SDS* aggregation and stacking modes, which transfer from huge lamella micelle in solution to isotropic structure in solid patterns.

As for the latter aspect, the molecular assembly process is closely related to molecular structure characteristics, such as symmetric and asymmetric ones, which will lead to discrepancies in aggregation evolution, packing mode, and reconfiguration capability. The spatial polarization profile of light emission (*29*) and grazing incidence wide-angle x-ray scattering (GIWAXS) are performed to characterize the in-plane and out-of-plane molecular packing modes, respectively. It is observed that the line of symmetric *TPE-diSDS* shows fluorescence orientation at an angle of about 70° to its long axis, while the linking node of multiple lines exhibits isotropy (Fig. 5B and fig. S13, A to C). The in-plane packing mode is also confirmed by calculated results of the molecular transition dipole moment (Fig. 5C, fig. S13D, and table S2). In GIWAXS, a sharp peak is detected in out-of-plane direction and consistent with the x-ray diffraction (XRD) results (Fig. 5A and fig. S14D), indicating the highly ordered and layered packing mode. GIWAXS of asymmetric *TPE-SDS* shows isotropic signals (fig. S15), indicating that it is no longer characteristic of a well-layered stacking. The results reveal that the BTMP method can induce molecular ordering at the micro/nanoscale with desirable structural characteristics. Furthermore, the molecular dynamics (MD) simulation of the BTMP process shows the difference in the assembly behavior of different molecules near the dried state (movie S2). Moreover, *TPE-diSDS* with symmetric structure exhibits good reconfiguration capability with the contraction of bubble wall confinement space and keep a stable lamella stacking mode during the assembly process, ranging from wet state, drying state, to dried state (Fig. 5D, top). The hydrophilic fragments point to both sides of the lamellar, with a thickness of about 2 nm. On the other hand, asymmetric *TPE-SDS* shows poor ability to motion and reconfiguration. The initial ordered structure gradually evolves into isotropic structure like vesica with the drainage and evaporation (Fig. 5D, bottom). Considering the above results together with solution cryo-TEM and small-angle neutron scattering (SANS) data (fig. S16), we propose a confinement-induced aggregation and assembly mechanism (Fig. 5, E and F). In the middle part of the bubble wall, symmetric molecules tend to aggregate into small spherical micelle (radius about 1 to 2 nm; fig. S16, A and C) in solution and assemble to an ordered in-plane lamellar structure and out-of-plane layered structure accompanied with the contraction of bubble wall thin liquid film. The asymmetric hydrophilic-hydrophobic structure leads to molecular aggregation and formation of a huge lamella micelle (thickness about 3.14 nm and length about 100 to 200 nm; fig. S16, B and D) in solution, which becomes compressed and folded in the following drying steps and constructs an isotropic structure eventually.

The results demonstrate that the symmetry plays a great role in deciding molecular aggregation size and evolution process in the solution and the molecular motility and reconfiguration capability during the continuous thinning procedure of the soft confinement space as well. Molecules with symmetric structures are favorable to form smaller aggregates, stronger reconfiguration ability, and more ordered molecular patterns, reaching the few-layer precision. On the contrary, the ones with asymmetric structures can form larger aggregates, weaker reconfiguration ability, and less ordered patterns, making the thin liquid film rupture in advance and only realize 50-nm precision. In the perspective of real molecular-scale precision near Newton black film, it is worth noting that the ordering of the interface phase will play a more apparent influence rather than that of the bulk phase, which is dominant for the current precision level. Therefore, because asymmetric molecules favor denser and ordered molecular film at the gas-liquid interface in the MD simulation and L-B film results (fig. S6, C to E), it is expected to achieve the ultimate Newton black film thickness in the future.

## DISCUSSION

We have developed an efficient bubble-template printing method for the fabrication of nano- and molecular-scale patterns. Using the template of the bubble arrays, we can precisely confine the molecular assembly and achieve various morphologies with sub–12 nm precision. In particular, we demonstrate that the highest precision of BTMP could be predicted by the disjoining pressure isothermal curve, and the exact value is closely correlated with the molecular structure. In addition, we clarify the confinement-induced molecular aggregation and assembly mechanism for molecules with different symmetries. It is revealed that symmetric molecules with better reconfiguration capacity, smaller aggregates, and anisotropic lamellar structure are more favorable to construct the few-layer level molecular patterns. However, because the ordering of the interface phase will play a more apparent influence rather than that of the bulk phase when it comes to the Newton black film precision, the asymmetric molecules hold more hope for approaching the ultimate bilayer precision. These findings afford powerful solutions for ultrahigh-precision molecular patterning and provide promising opportunities for the fabrication of nano- and molecular-scale devices, especially high-sensitivity sensors (fig. S17), photoelectric devices with unique nanoconfinement and performance enhancements, and bioengineered tissues.

## MATERIALS AND METHODS

### Materials

The model molecules of *TPE-diSDS* and *TPE-SDS* were synthesized referring to earlier reports (*30*, *31*).

### Fabrication of molecular patterns by the bubble-template printing method

The processing of the template with columnar microstructure has been described in the previous article (*24*). In the process of molecular pattern printing, 1.5 μl of 0.3% (weight/weight) sodium borohydride ethanol solution was dropped on the side of the template, and an equal volume of *TPE-diSDS* solution with a certain concentration was dropped to the cleaned surface of the substrate. After assembling the template and the substrate to form a sandwich structure, it was left for 4 hours at room temperature. After separating the template, high-precision molecular patterns could be obtained on the surface of the substrate. The CA of substrate and template in our experiment was controlled by adjusting plasma treatment time and power. As for the formation process of other line morphologies, the formation of broken line 1 is similar to the inhomogeneous line, both of which are due to the bubble rupture on the template side and the additional pinning of the template. The difference is that the former does not have enough molecules to realize the continuous line. The dominant reason for broken line 2 and bead line is the CA of the substrate. The high CA (CA ≥ 90°) limits the shrinkage of liquid into the gap between the template and the substrate, thus forming these line morphologies affected by the template. Besides, the broken line 2 also requires low concentration to realize discontinuous morphology.

### Characterization

The structure of the molecular patterns was investigated by SEM (SU-8020 and S-4800) and TEM (JEM-1011). Optical microscopy images and cross-polarized optical microscopy were obtained by Nikon (ECLIPSE LV100 POL, Japan). Static CAs were measured on a DataPhysics OCA20 CA system at the substrate. The experimental results of fluorescence visualization and temperature sensor were completed using confocal microscope C2 and A1 produced by Nikon Company. The 3D image results were achieved by layer-by-layer scanning and 3D reconstruction. In addition, the wavelength of exciting light was 405 nm, and the detection wavelength of 450 to 600 nm was selected. White light interferometric imaging of printed molecular patterns was characterized by surface profile instrumentation from Bruker Company. AFM results were also achieved with Bruker’s Multimode 8 AFM. Cu Kα source x-rays was used in XRD (Empyrean, Panalytical). The spatial polarization profile of light emission for the solid patterns was characterized by the excitation of mercury lamp and the detection of luminescence information from different angles by the linear polarizer. Nuclear magnetic resonance spectra were recorded by Bruker Avance (400 MHz) spectrometer. Mass spectrum was measured on matrix-assisted laser desorption/ionization–time-of-flight liquid chromatography–mass spectrometry of Bruker. Fluorescence spectra were taken on a Horiba spectrofluorometer (FluoroMax), and the excitation wavelength was set to 310 nm. Ultraviolet-visible (UV-vis) spectrum was taken on a UV-vis spectrophotometer (Lambda 1050, PerkinElmer). Surface tension of the solutions was measured through Wilhelmy hanging plate method and BZY-2 tensiometer.

GIWAXS were measured on a Xeuss 2.0 SAXS/WAXS system (Xenocs SA, France). Cu Kα x-ray source (GeniX3D Cu ULD), generated at 50 kV and 0.6 mA, was used to produce x-ray radiation with a wavelength of 1.5418 Å. A semiconductor detector (Pilatus 300 K, DECTRIS, Swiss) with a resolution of 487 × 619 pixels (pixel size = 172 μm by 172 μm) was used to collect the scattering signals. The incident angle was 0.3°. Each WAXS pattern was collected with an exposure time of 30 min. The sample-to-detector distance was 137.5 mm, which was determined by a silver behenate (AgC_22_H_43_O_2_) standard. The 1D intensity profiles were integrated from background-corrected 2D WAXS patterns.

The disjoining pressure isothermal curve was measured by a homemade Scheludko-like cell (*22*) combined with the in situ–reflected light microscope illuminated by white light. The thin liquid film was formed in a hole at the center of a porous glass plate, which was sealed in a closed container. The liquid film thinned along with boosting the container pressure and drainage from the film into the meniscus, porous plate, and capillary tube that connected with the plate. The disjoining pressure was calculated on the basis of the following expression (*22*)

$$\pi =P_{g}-P_{c}-\rho gh+\frac{2\gamma }{r}$$

where π is the disjoining pressure in a specific film thickness, *P_g_* is the air pressure in the closed cell, *P_c_* is the ambient pressure, ρ is the solution density, *g* is the gravitational acceleration, *h* is the liquid height in the capillary tube, γ is surface tension, and *r* is inner diameter of capillary. The thickness of the film was calculated by interferometry digital imaging optical microscopy protocols (*27*) based on the microphotograph and the following expression

$$h=\frac{\lambda }{2n\pi }\sin^{-1}\left(\sqrt{\frac{\varphi }{1+4R(1-\varphi )/{\left(1-R\right)}^{2}}}\right)$$

$$\varphi =(I-I_{\min})/(I_{\max}-I_{\min})$$

where *h* is the thickness of film; λ is the wavelength of light after reading a photo as a composite of red (630 nm), green (546 nm), and blue (470 nm); *I* is the intensity measured in each pixel; *I*_max_ and *I*_min_ are the maximum and minimum intensity values; *n* is the refractive index; and *R* is the Fresnel coefficient of the solution. Increasing the pressure inside the cell to change the thickness of film can thereby get the entire disjoining pressure isotherm curve.

Cryo-TEM images were acquired by a Themis 300 (Thermo Fisher Scientific). For the sample preparation, a total of 3 μl of solution were deposited onto a holey carbon film on copper grid [grid in grid (GIG)] and flash-frozen in liquid ethane cooled down at liquid nitrogen temperature using a Vitrobot Mark IV (Thermo Fisher Scientific) system. The vitrified samples were then transferred to a 626 cryogenic sample holder (Gatan) and examined with the cryo-TEM at 77 K. Micrographs were captured with a Falcon III camera (Thermo Fisher Scientific).

Samples used for SANS measurements were prepared in deuterated water to maximize the scattering contrast between solvent and molecules. Spherical model with hard sphere interference and lamella model were used in the data fitting according the feature of the samples.

### Simulations

Electrostatic double-layer force was calculated according to simplified constant surface potential mode (*22*) and following expression

$$F_{\mathrm{ele}}=64n^{0}kT\gamma^{2}e^{-\kappa h}$$

$$\gamma =\frac{e^{\frac{e\psi }{2kT}}-1}{e^{\frac{e\psi }{2kT}}+1}$$

$$\lambda =\frac{1}{\kappa }=\sqrt{\frac{\varepsilon kT}{8\pi n^{0}e^{2}}}$$

where *n^0^* is number density of ions, *k* is the Boltzmann constant, *T* is the temperature, *h* is the film thickness, *e* is the elementary charge, ψ is the surface potential, and ε is the dielectric constant of the solution. The London–van der Waals contribution was computed using the following expression (*32*)

$$F_{\mathrm{van}}=-\frac{A}{6\pi h^{3}}$$

where *A* is the Hamaker’s constant and *h* is the film thickness.

Density functional theory (DFT) calculations were performed with the Gaussian 09 D.01 program package (*33*). Geometry optimizations were conducted at the B3LYP-D3(BJ)/6-31G** level of theory, and all optimized structures indicated no imaginary frequency. Vertical transitions were calculated using TD-DFT (PBE0/6-311+G**). The transition dipole moment is visualized with multiwfn (*34*) and VMD (*35*). To simplify the calculation and avoid the influence of strong polar groups on the transition dipole moment, we simplified the structure of *TPE-diSDS* by removing the sulfonate groups.

All-atom MD simulations were performed with the Gromacs package 4.5.4 (*36*). CharmM36 force field was used to model the *TPE-diSDS* and *TPE-SDS* molecules (*37*). Water molecules were modeled using the tip3p potential. MD simulation of the 2D liquid foam layer system consisted of 90 *TPE-diSDS* or *TPE-SDS* in a box sized 4.2 nm by 35.0 nm by 3.0 nm. The wet layer system consisted of 2400 water molecules and 244 chloride ions, and the charge was counterbalanced by adding 424 and 334 sodium ions for *TPE-diSDS* and *TPE-SDS*, respectively. The *TPE-diSDS* and *TPE-SDS* molecules were initially packed referring to our previous simulation work of meso-tetra(4-sulfonatophenyl) porphyrin (TPPS) (*25*). Both the wet and dry layer systems were first minimized using the steepest-descent method and the conjugate-gradient algorithm until it converged. The solvent was then relaxed for 100 ps at 313 K, with the positions of the *TPE-diSDS* and *TPE-SDS* atoms restrained by a harmonic potential. Bond lengths were constrained by the LINCS algorithm (*38*). The electrostatic interactions were calculated using the particle mesh Ewald algorithm with a cutoff of 1.0 nm (*39*). The cutoff radius for the Lennard-Jones interactions was set to 1.0 nm. A dielectric constant of 1 and a time step of 2 fs were used. All simulations were performed using the constant NVT ensemble. The temperature of the system was kept constant at 313 K using the Nosé-Hoover thermostat with a time constant of 0.4 ps (*40*). To desolvate, the interaction of the water molecules was turned off gradually in the first 1 ns in the dry layer simulation.

## Supplementary Materials

### This PDF file includes:

Legends for movies S1 and S2 Tables S1 and S2 Figs. S1 to S17 References
