# Supplementary material for: Bubble wall confinement–driven molecular assembly toward sub–12 nm and beyond precision patterning
Source: Sci Adv. 2023 Mar 15;9(11):eadf3567. doi: 10.1126/sciadv.adf3567 (PMC10017045; doi:10.1126/sciadv.adf3567)
Supplement: Supplementary file 1 — Legends for movies S1 and S2 Tables S1 and S2 Figs. S1 to S17 References [file sciadv.adf3567_sm.pdf]

Science Advances
AAAS

Supplementary Materials for

Bubble wall confinement–driven molecular assembly toward sub–12 nm and beyond precision patterning

Zhiyuan Qu *et al.*

Corresponding author: Xuehai Yan, yanxh@ipe.ac.cn; Yali Qiao, qiaoyl@iccas.ac.cn;
Yanlin Song, ylsong@iccas.ac.cn

Sci. Adv. **9**, eadf3567 (2023)
DOI: 10.1126/sciadv.adf3567

The PDF file includes:

Legends for movies S1 and S2
Tables S1 and S2
Figs. S1 to S17
References

Other Supplementary Material for this manuscript includes the following:

Movies S1 and S2

Supplementary Movies

Movie S1 Bubble rupture process in bubble-template molecular printing method.

Movie S2 Molecular assembly process in bubble-template molecular printing method.

Supplementary Tables

Table S1 The precision of other patterning methods for organic materials.

Method	Common Precision Range	Record Precision	Application	Ref.
Edge lithography (E-L)	Sub-100 nm	60 nm	Mask for etching	41
		30 nm	Photoresist patterning	42
		50 nm	SAM patterning	43
		7.5 nm	TiO_2 patterning	44
Liquid bridge self-assembly (LBA)	Sub-100 nm	30 nm	Polymer patterning	45
		100 nm	Calcein nanowire arrays for ferrous salt sensing device	46
		48 nm	Nanoparticles patterning for optical waveguiding properties	47
Scanning probe lithography (SP-L)	Sub-50 nm	15 nm	Molecular monolayers	12
Nanoimprinting lithography (NI-L)	Sub-10 nm	8 nm	Nanofabrication	15
		10 nm	Thermal resist patterning on the lens surface	48
		10 nm	-	13
Block copolymer self-assembly (BCPA)	Sub-10 nm	6 nm	Template for nanoparticles assembly	18
		10 nm	Copolymer patterning	49
DNA origami lithography (DNAo-L)	Sub-10 nm	5 nm	Single-molecular emitter	50
		60 nm	Single nanoparticle chain waveguides	51

Table S2 Coordinates of simplified *TPE-diSDS* at B3LYP-D3 (BJ)/6-31G** level of theory.

TPE-diSDS							
Element	Coordinates (angstroms)			Element	Coordinates (angstroms)		
	X	Y	Z		X	Y	Z
C	-4.62071	-0.12267	-0.56596	H	-2.37266	-1.64204	1.47193
C	-4.12487	-0.94168	0.45445	H	-1.68546	1.14919	-1.70824
C	-2.75032	-0.99794	0.68427	H	-4.1278	1.24101	-2.14437
C	-1.84474	-0.23937	-0.06803	H	-0.91139	-2.54743	-1.23896
C	-2.36555	0.56413	-1.09961	H	-0.03661	-4.83594	-0.87895
C	-3.72696	0.62479	-1.34687	H	1.49921	-5.29695	1.02227
C	-0.38533	-0.31529	0.19815	H	2.14748	-3.44716	2.5541
C	0.14862	-1.68684	0.42331	H	1.29581	-1.15793	2.16353
C	0.41447	0.79128	0.23166	H	2.16772	2.18575	1.76298
C	1.89729	0.70641	0.21883	H	4.64884	2.03642	1.76378
C	-0.1403	2.17152	0.28267	H	4.4545	-0.88572	-1.38121
C	-0.23125	-2.74558	-0.41679	H	2.00697	-0.75279	-1.35354
C	0.25854	-4.03336	-0.20939	H	-1.5744	1.74267	1.82638
C	1.12154	-4.29256	0.8563	H	-2.46289	4.04945	1.92976
C	1.4876	-3.25305	1.71373	H	-1.54376	5.80755	0.42969
C	1.0057	-1.96489	1.49997	H	0.29423	5.24001	-1.14654
C	2.66945	1.50507	1.08279	H	1.19851	2.93558	-1.21777
C	4.05373	1.43119	1.08843	H	-6.82034	-0.467	0.93647
C	4.71607	0.56367	0.20842	H	-6.6925	-1.80923	-0.21836
C	3.96889	-0.21993	-0.67881	H	-8.30343	-0.64226	-1.73722
C	2.57777	-0.13962	-0.66522	H	-8.43015	0.68521	-0.59014
C	-1.16812	2.51007	1.1771	H	-9.33983	-0.90337	1.1423
C	-1.66891	3.80765	1.22947	H	-9.21705	-2.22569	-0.00571
C	-1.15118	4.79602	0.38992	H	-10.8669	-1.0662	-1.52579
C	-0.11905	4.47706	-0.49345	H	-10.9911	0.26381	-0.36866
C	0.38817	3.18025	-0.53855	H	6.58678	-0.05636	-1.62938
O	-5.94014	0.00812	-0.88307	H	6.4939	-1.34732	-0.41444
C	-6.895	-0.73204	-0.1282	H	8.45749	-0.35669	0.77666

C	-8.27498	-0.39751	-0.66864	H	8.54792	0.92455	-0.42531
C	-9.38835	-1.14383	0.07194	H	8.97104	-0.79155	-2.22016
C	-10.7807	-0.80687	-0.46499	H	8.8807	-2.07023	-1.02083
O	6.07715	0.56196	0.28618	H	10.88104	-1.09854	0.17451
C	6.80319	-0.30521	-0.5803	H	10.97286	0.18784	-1.03396
C	8.28253	-0.12979	-0.28184	H	-11.5604	-1.35097	0.07676
C	9.16415	-1.01898	-1.16341	H	11.26645	-1.49149	-1.50695
C	10.65534	-0.84727	-0.86752	H	-4.79103	-1.53576	1.0674

Supplementary Figures

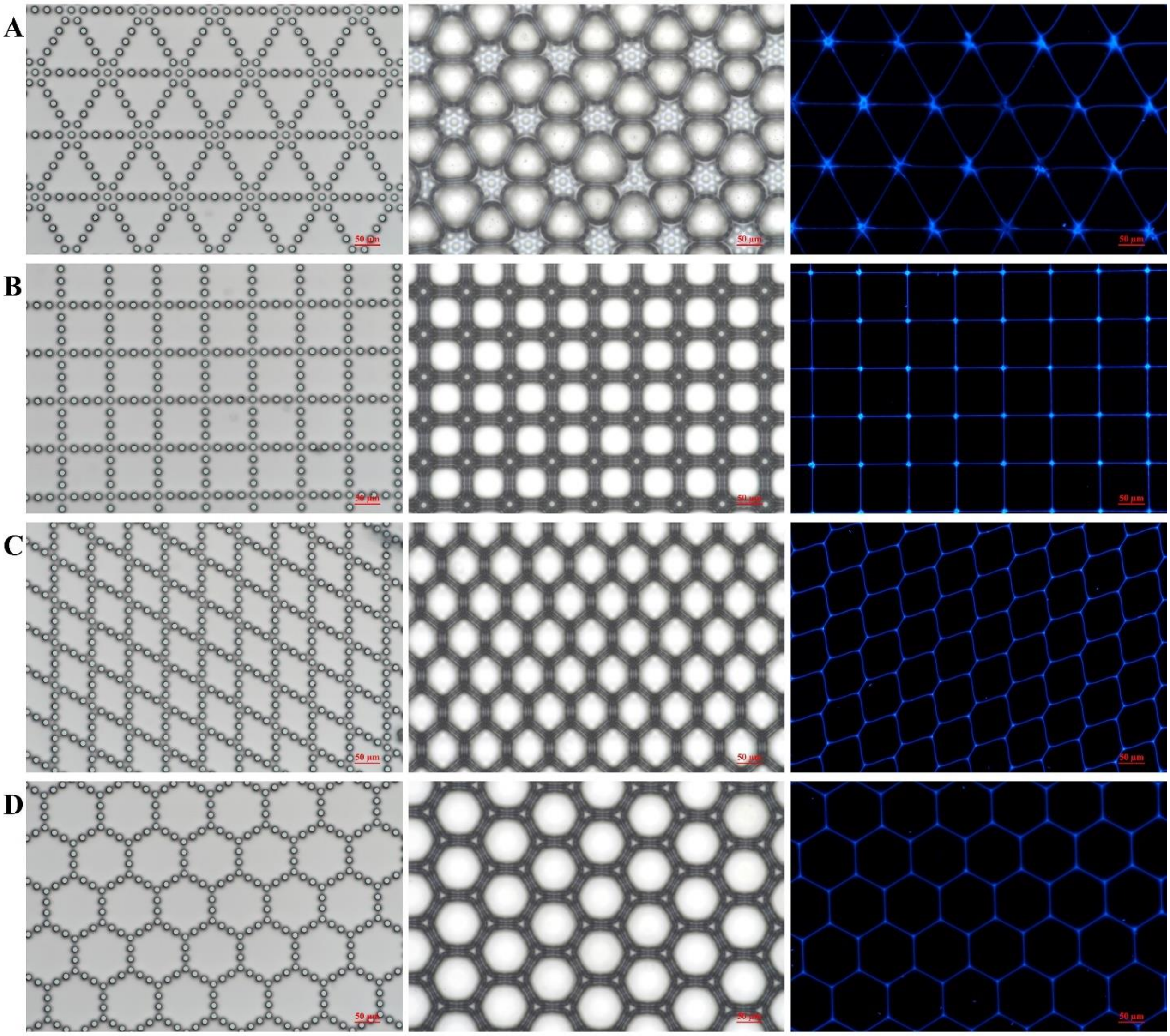

Fig. S1. Printed molecular patterns with various topological structure, including triangle (A), square (B), rhombus (C) and hexagon (D). The left image is the template with columnar microstructure, the middle image is corresponding bubble arrays, and the right image is printed molecular patterns under fluorescence microscope.

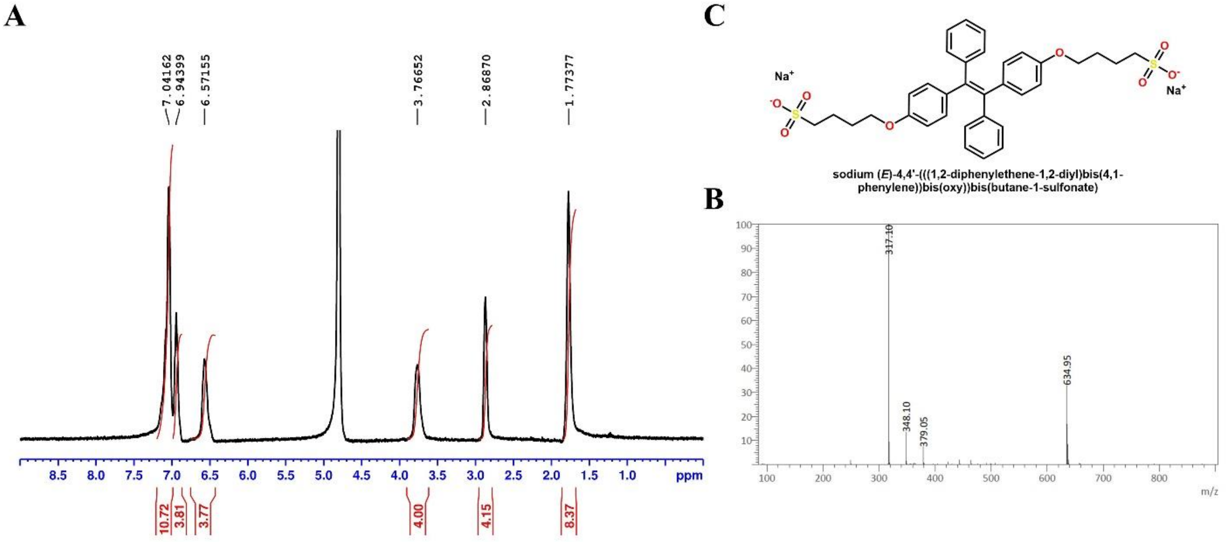

Fig. S2. Characterization of model molecular structure. (A) ^{1}H NMR and **(B)** MS spectrum of *TPE-diSDS*. **(C)** Structure and IUPAC name of *TPE-diSDS*.

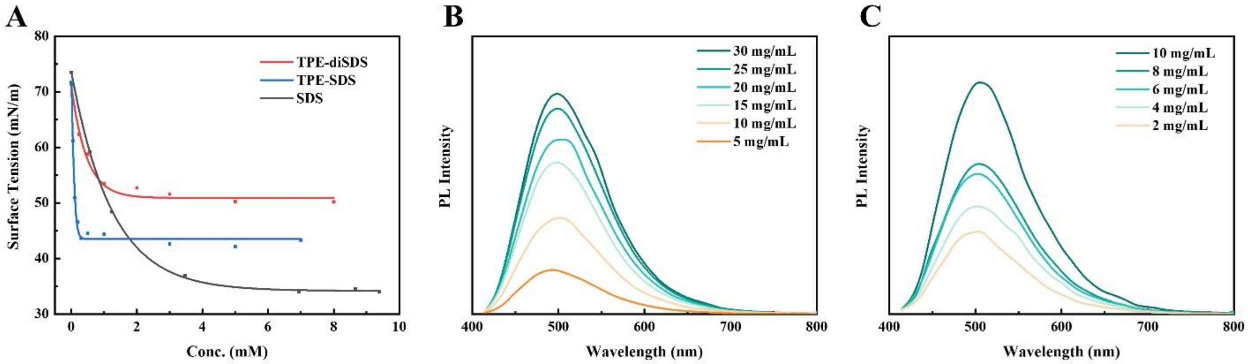

Fig. S3. Functional integration of the two-fragment molecular design strategy. (A) The surface activity characteristic of *TPE-diSDS* and *TPE-SDS* when pH=2. The reference curve is sodium dodecyl sulfate (*SDS*). It should be noted that although the model molecules (*TPE-diSDS* and *TPE-SDS*) both have surface activity, the concentration used in our experiment is higher than the critical micelle concentration of these molecules (CMC = 1.5 mM for *TPE-diSDS* and 0.3 mM for *TPE-SDS*), so the solution concentration and contact angle of substrate are two independent parameters. **(B)** AIE properties of *TPE-diSDS* in the range of experimental concentration (from 5 mg/mL to 30mg/mL). **(C)** AIE properties of *TPE-SDS* from 2 mg/mL to 10 mg/mL.

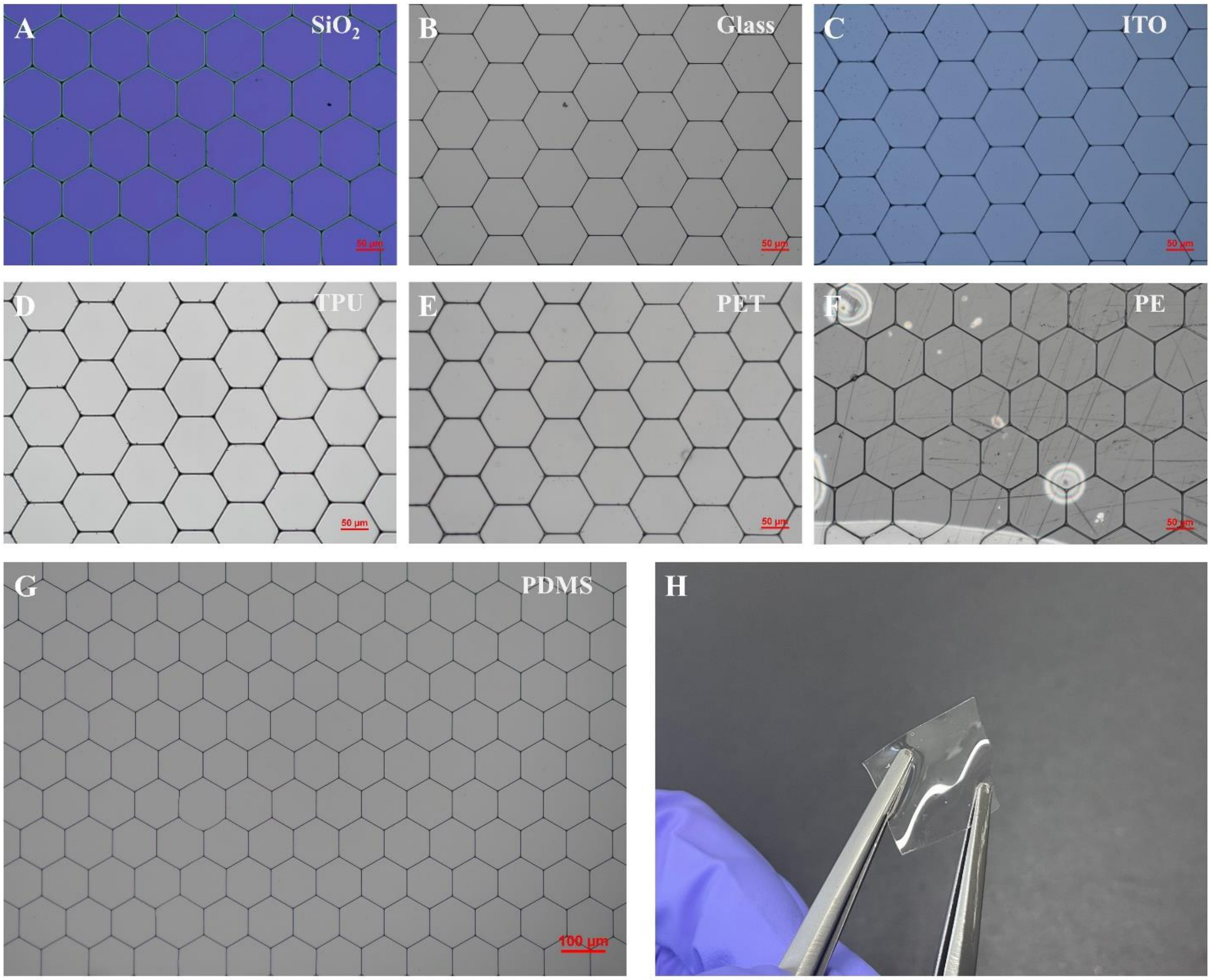

Fig. S4. Printed molecular patterns on various substrates based on BTMP method, including rigid substrates (**(A)-(C)** silicon dioxide, glass, and indium tin oxide) and flexible substrate (**(D)-(F)** thermoplastic polyurethane, polyethylene terephthalate and polyethylene). **(G)** and **(H)** are printed molecular patterns on polydimethylsiloxane (PDMS).

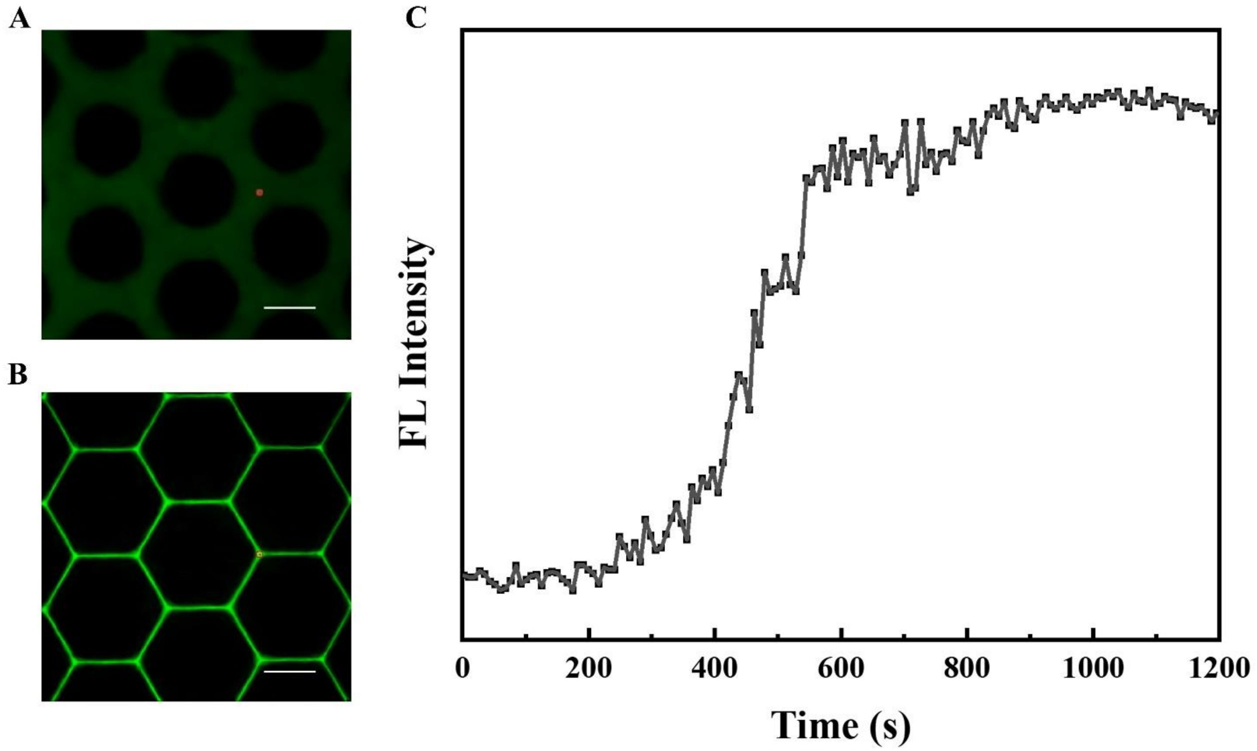

Fig. S5. The visualization of BTMP process by confocal microscope, excited by 405 nm laser and detected emission from 400 nm to 600 nm. The initial state **(A)** and final state **(B)** of the printing process. **(C)** The time-varying fluorescence intensity curve of the whole printing process. The printing process of BTMP is an evolution from liquid to solid, and the AIE effects is conducive to the in-situ observation of this whole process due to the sufficient fluorescence intensity, especially for the later period of printing. Scale bars in **A** and **B** are 50 μm.

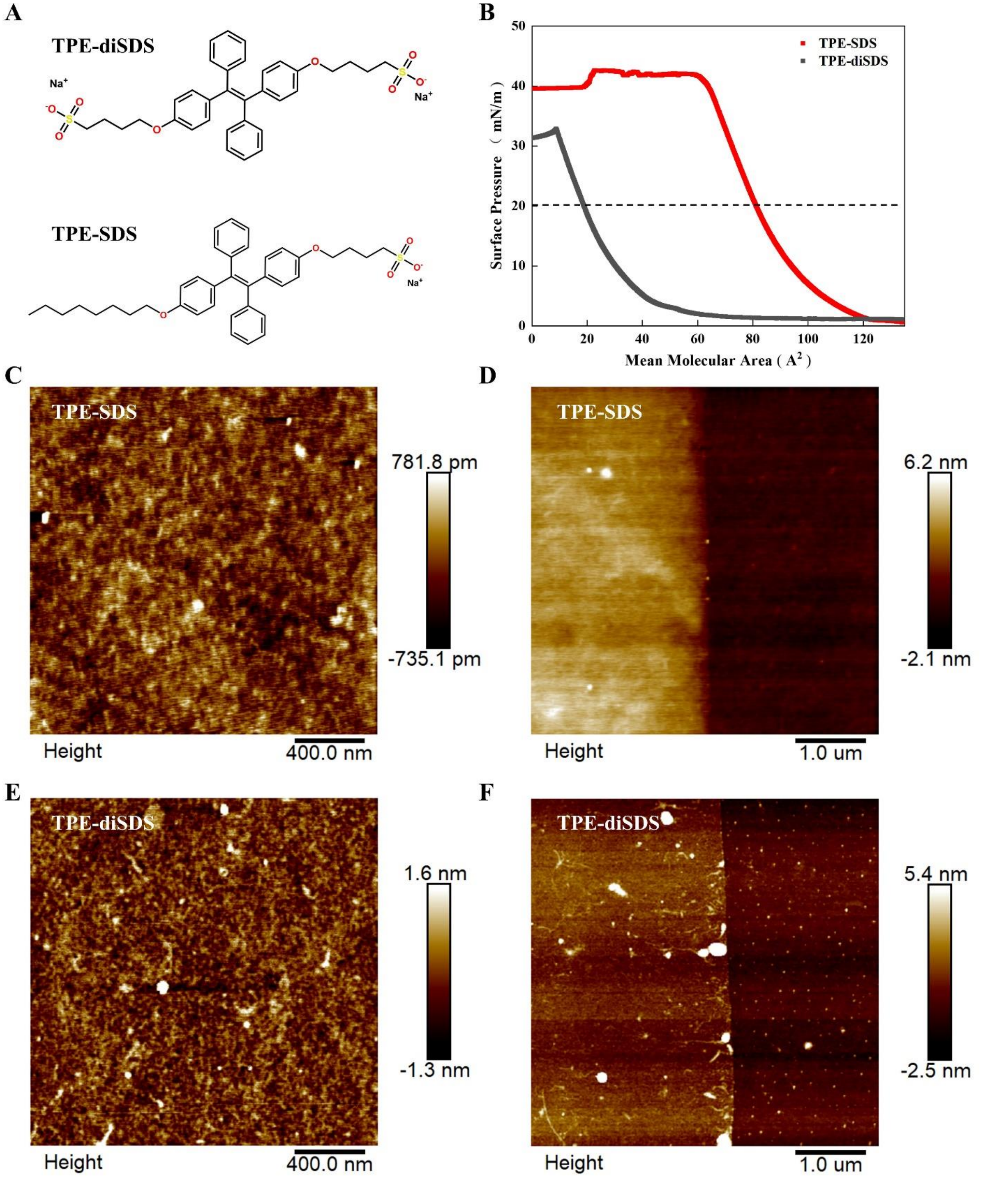

Fig. S6. The effect of the molecular symmetry on the air-liquid interface assembly. (A) Molecular structure of *TPE-diSDS* and *TPE-SDS*. **(B)** The surface pressure–mean molecular area curves of *TPE-diSDS* and *TPE-SDS*. The concentration of solution used to measure are both 0.5mM. **(C)-(F)** are AFM results of Langmuir-blogett membranes fabricated by *TPE-SDS* (**C** and **D**) and *TPE-diSDS* (**E** and **F**) when surface pressure is 20 mN/m. The former is flatter than the latter (roughness of **C** is 0.159 nm and **E** is 0.323 nm).

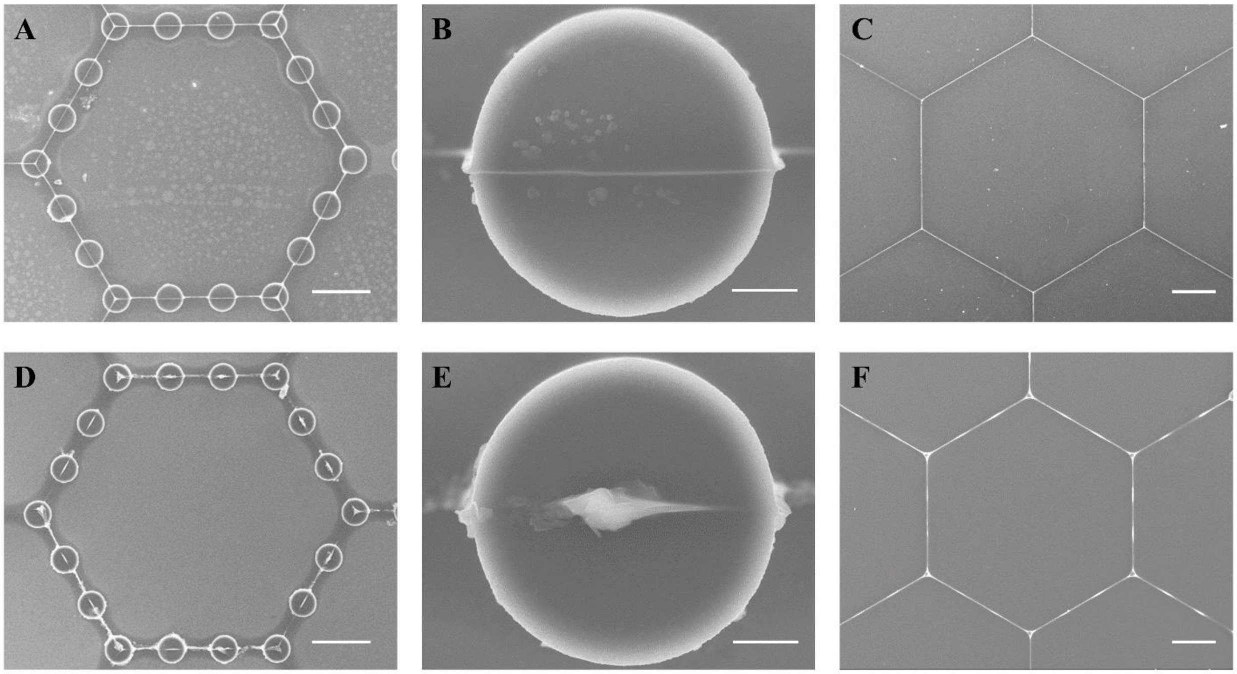

Fig. S7. The SEM results of uniform line (A-C) and inhomogeneous line (D-E). (A)-(B) and **(D)-(F)** are template side morphology. **(C)** and **(F)** are printing results in the substrate. Scale bars in **A**, **C**, **D** and **F** are 20 μm, **B** and **E** are 2 μm. In the uniform line condition, the residual molecular also forms a wire structure at the top of the silicon column. However, the silicon column corresponding to the inhomogeneous line forms the point structure with vertical molecular accumulation due to different rupture locations and additional pinning action.

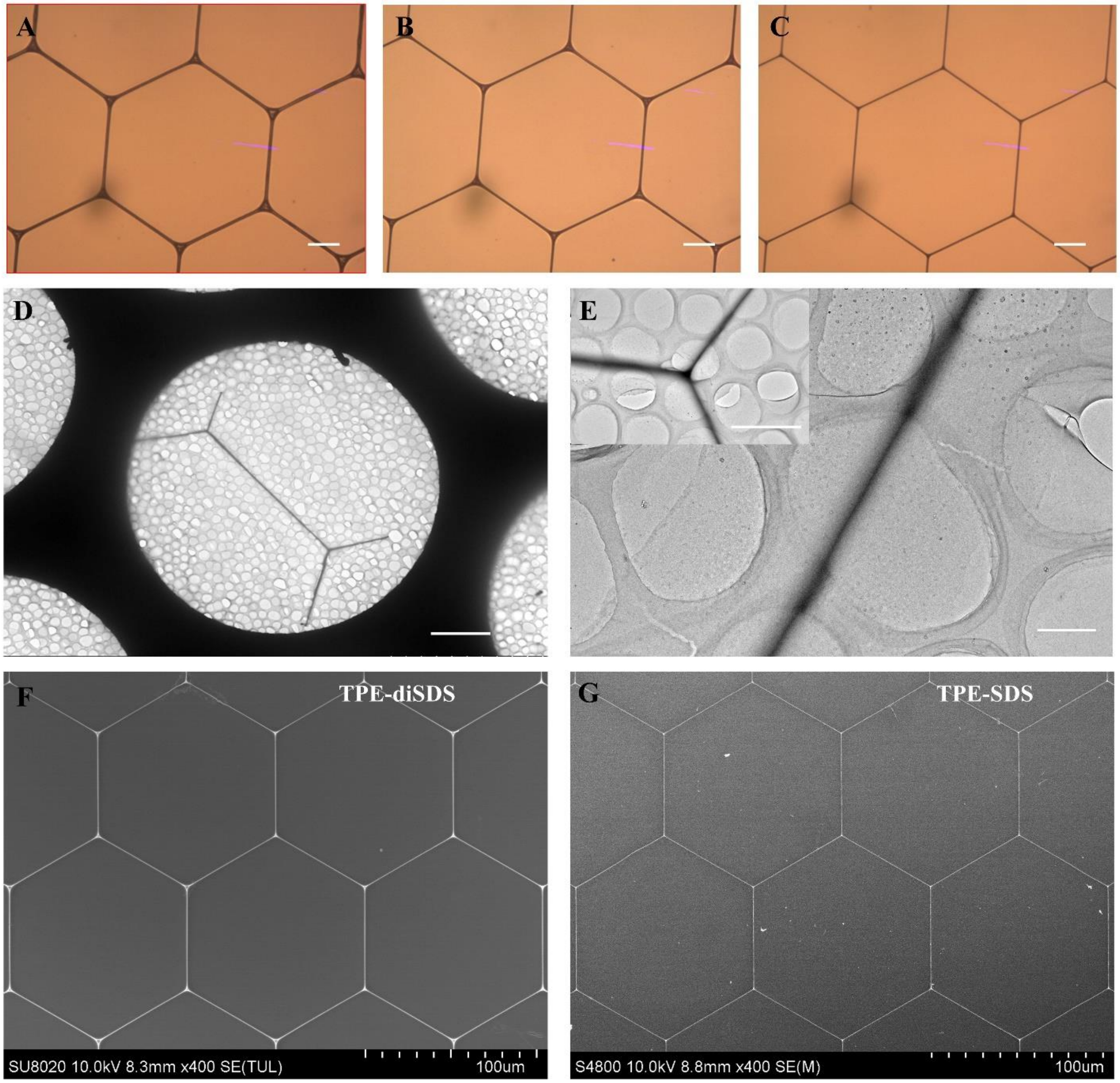

Fig. S8. Morphology of printed molecular patterns. **(A)**, **(B)** and **(C)** are optical microscope photographs of the grid-like patterns with different precision, including 110 nm **(A)**, 70 nm **(B)** and 30 nm **(C)**. **(D)** and **(E)** are TEM results of printed patterns. The dark areas are high-precision molecular patterns, with a thin molecular film underneath. **(F)** and **(G)** are SEM images of molecular patterns printed by different model molecules, **F** is *TPE-diSDS* and **G** is *TPE-SDS*. Scale bars in **A**, **B, C** and **D** are 20 μm, **E** are 1 μm.

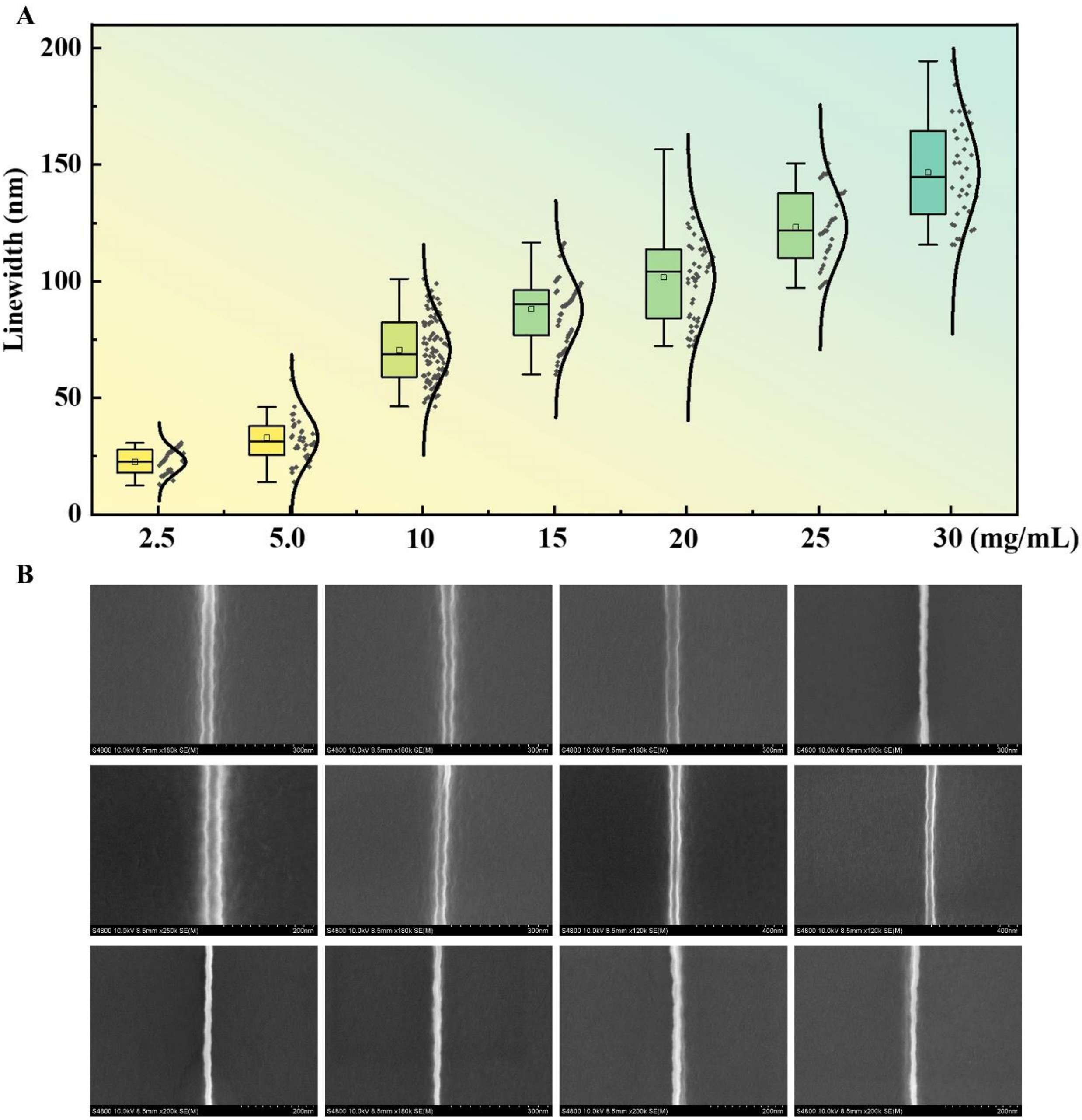

Fig. S9. The supplementary details of pattern precision statistics. (A) Distribution histograms of the line width in different solution concentration from 2.5 mg/mL to 30 mg/mL and specific contact angle (40°). **(B)** SEM images of *TPE-diSDS* molecular patterns in 5.0 mg/mL and CA=30°. The statistical results of this condition are associated with **Fig. 3C**. The line width is measured by SEM. In this work, 12-15 images in different positions of one experimental condition are recorded to measure the line width.

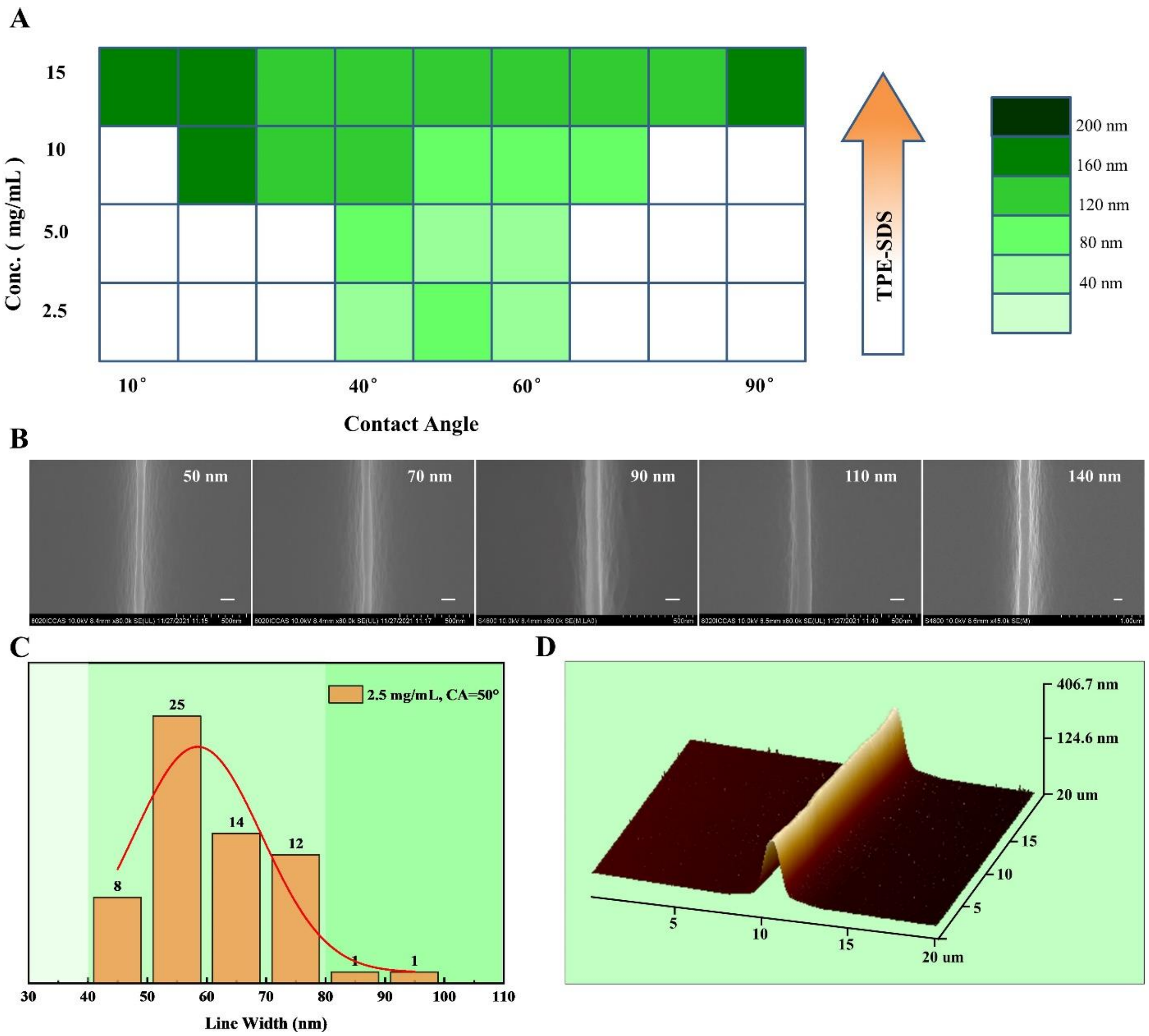

Fig. S10. Precision control of BTMP and *TPE-SDS*. (A) Phase diagram of various line width versus contact angle of the substrate and the concentration of solutions. **(B)** Scanning electron microscope images of molecular patterns with different feature sizes from 50 nm to 140 nm. Scale bars in **(B)** are 100 nm. **(C)** Distribution histograms of the line width in a special experiment condition, CA=50° and concentration=2.5 mg/mL. **(D)** The lateral morphology of a line in the patterns revealed by AFM.

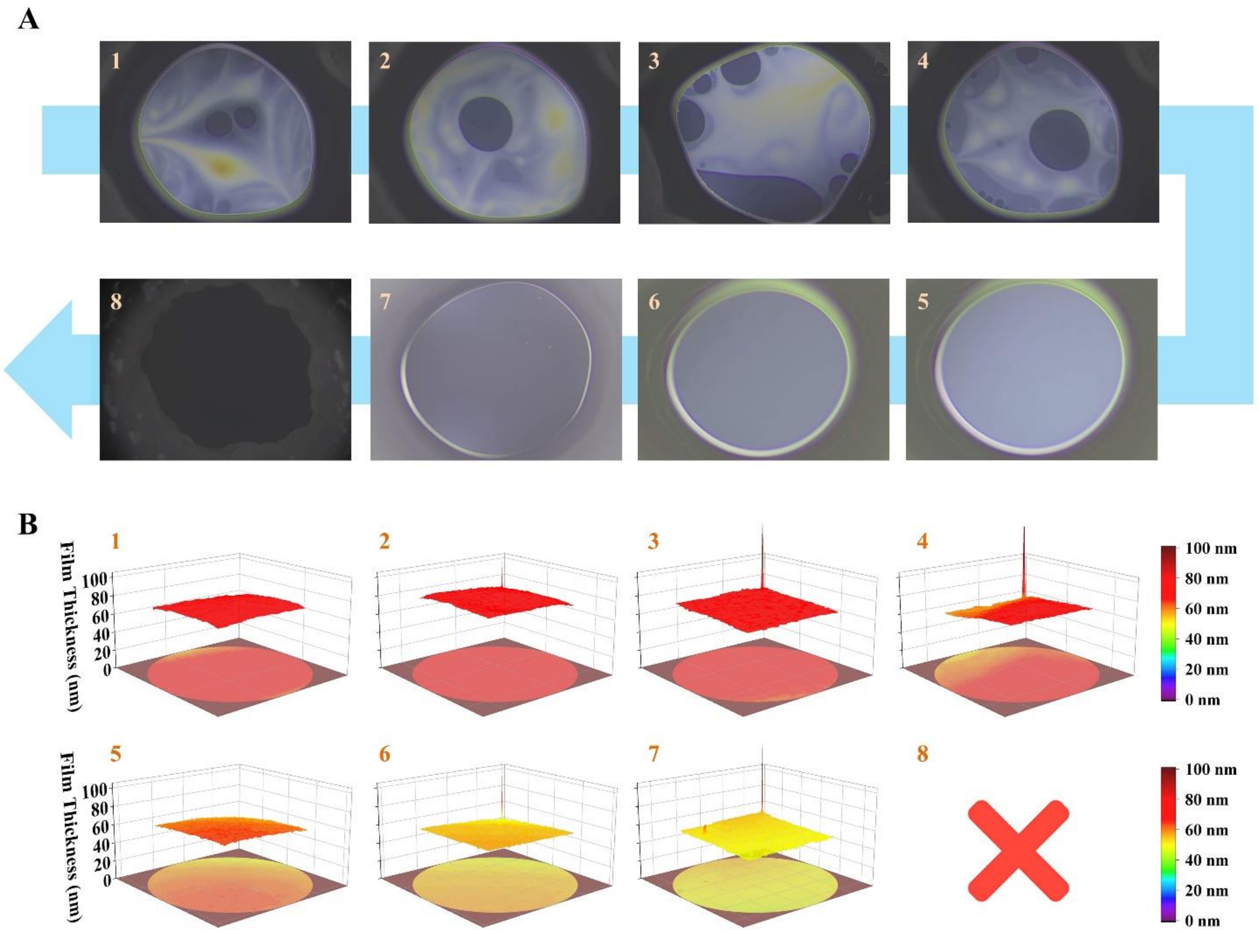

Fig. S11. The evolution and analysis of foam films constituted by *TPE-SDS*. (A) Foam film morphologies recorded by in-situ optical microscope. The liquid film is gradually drained and thinned from 1 to 8. **(B)** The corresponding thickness maps of foam film to **A**, which are calculated by IDIOM protocols.

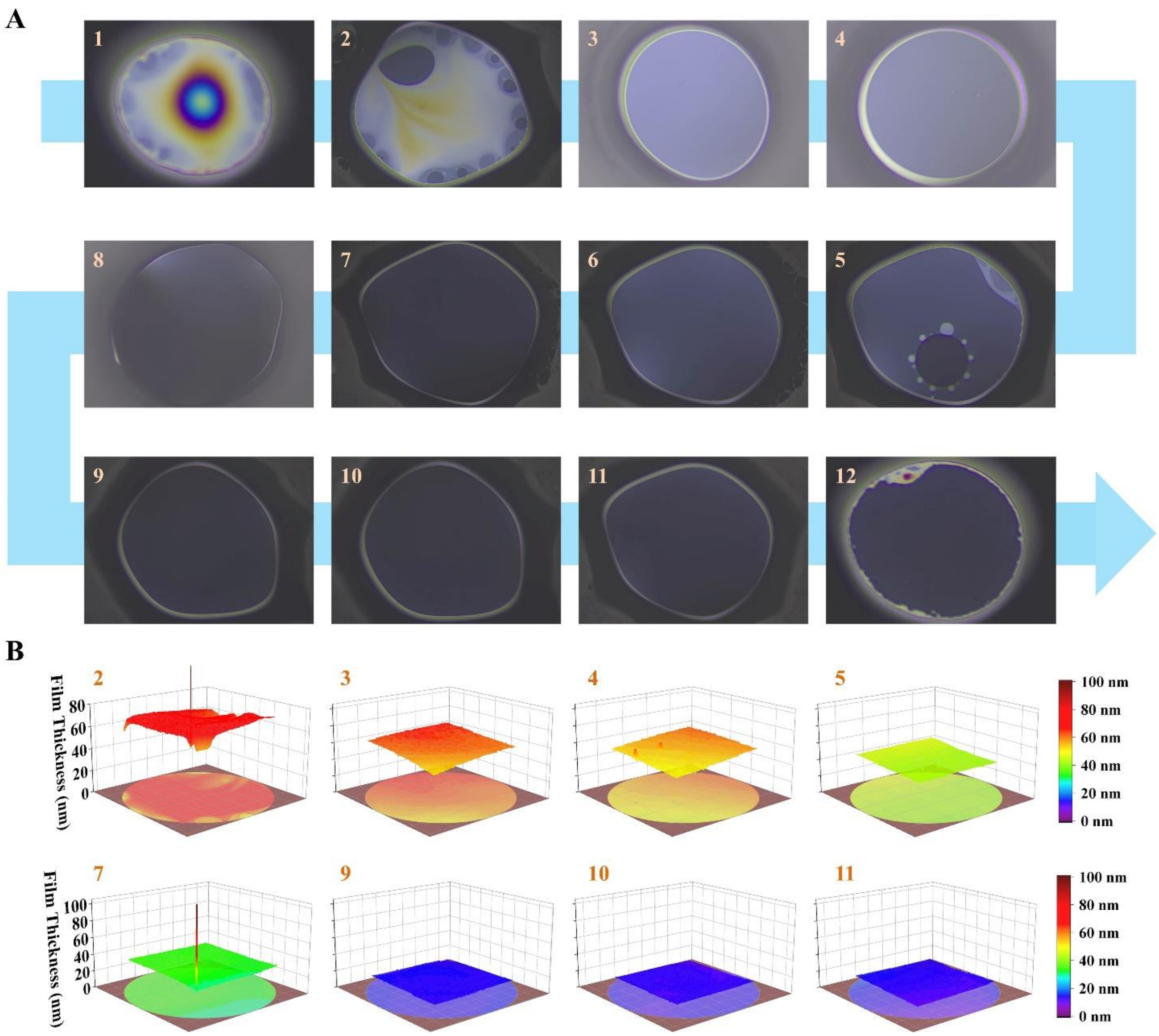

Fig. S12. The evolution and analysis of foam films constituted by *TPE-diSDS*. (A) Foam film morphologies recorded by in-situ optical microscope. The liquid film is gradually drained and thinned from 1 to 12. **(B)** The corresponding thickness maps of foam film to **A**, which are calculated by IDIOM protocols.

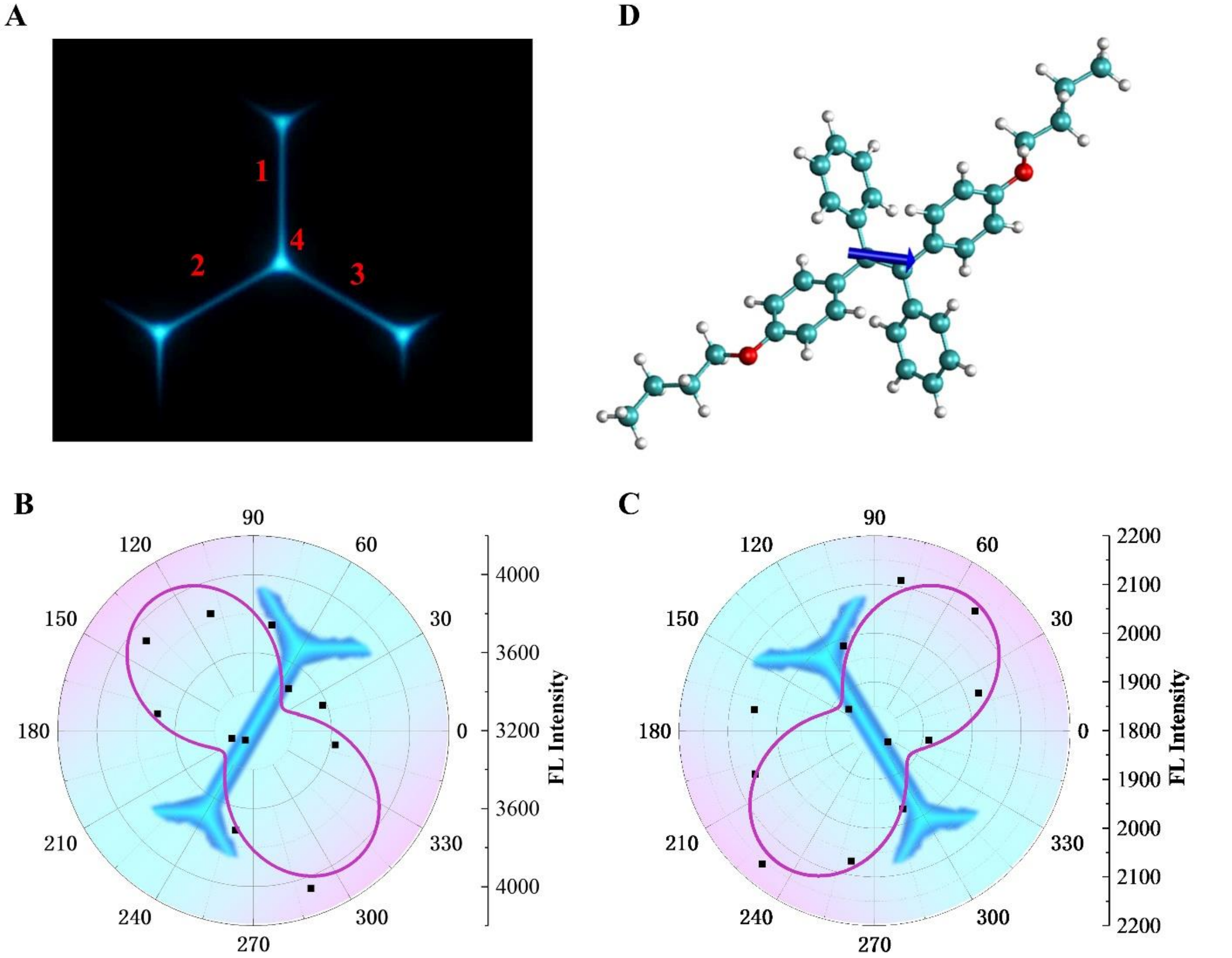

Fig. S13. The supplementary details of fluorescence microscopy images and spatial polarization profile of light emission tests. (A) Test location marker diagram of sample. Location 2 **(B)** and location 3 **(C)** both keep the same angle (70°) with line direction. **(D)** Structural illustration of optimized *TPE-diSDS* (simplified) with victor direction of transition dipole moment ($S_0 \rightarrow S_1$).

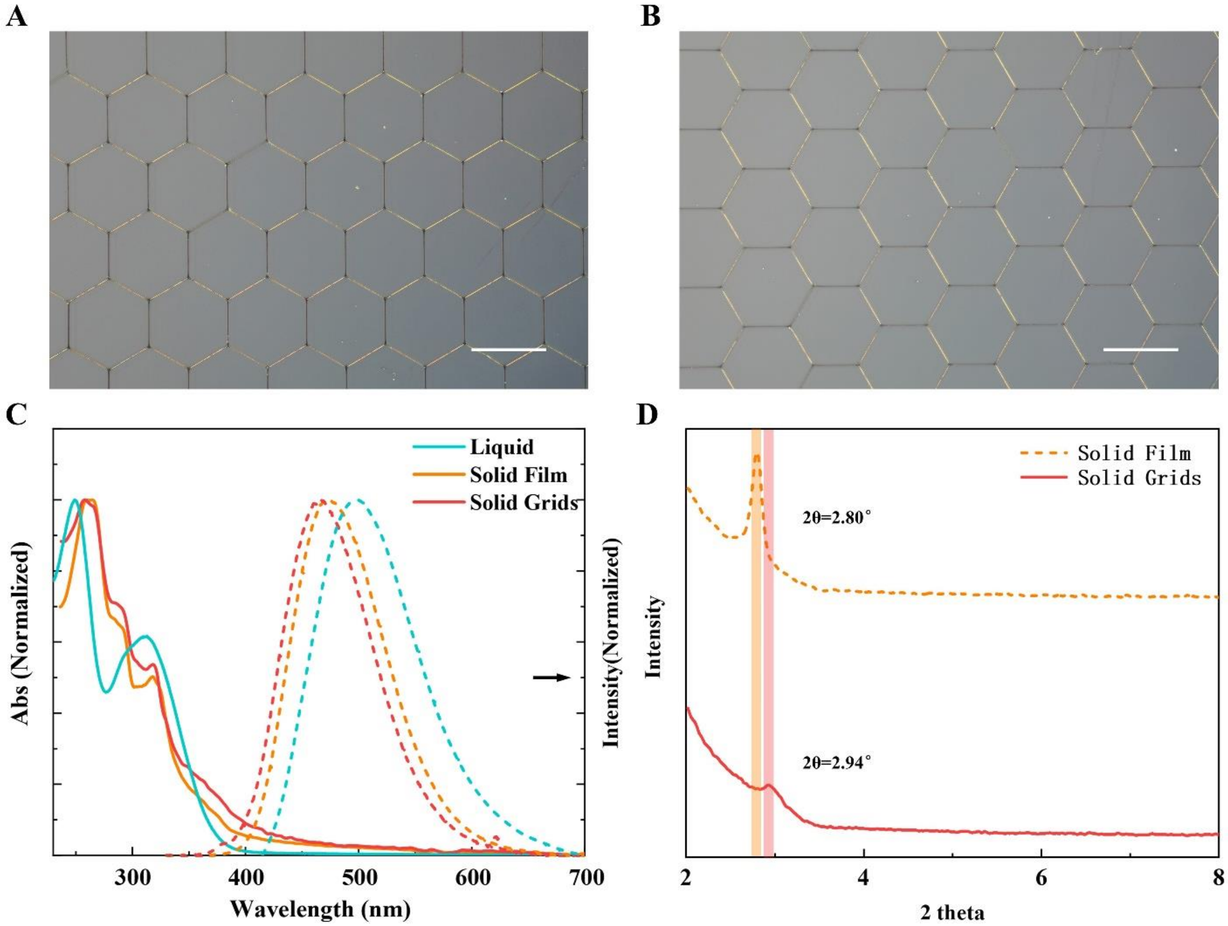

Fig. S14. More supplementary evidence for stacking ordering in molecular patterns. (A) and **(B)** are images of polarization microscope. UV-Vis spectrum, fluorescence emission spectrum **(C)**, excited by 310 nm) and X-ray diffraction results **(D)** of *TPE-diSDS*. Blue: *TPE-diSDS* solution when pH=2. Orange: *TPE-diSDS* solid film prepared by spin coating. Red: *TPE-diSDS* grids patten prepared by BTMP method. Scale bars in **A** and **B** are 100 μm.

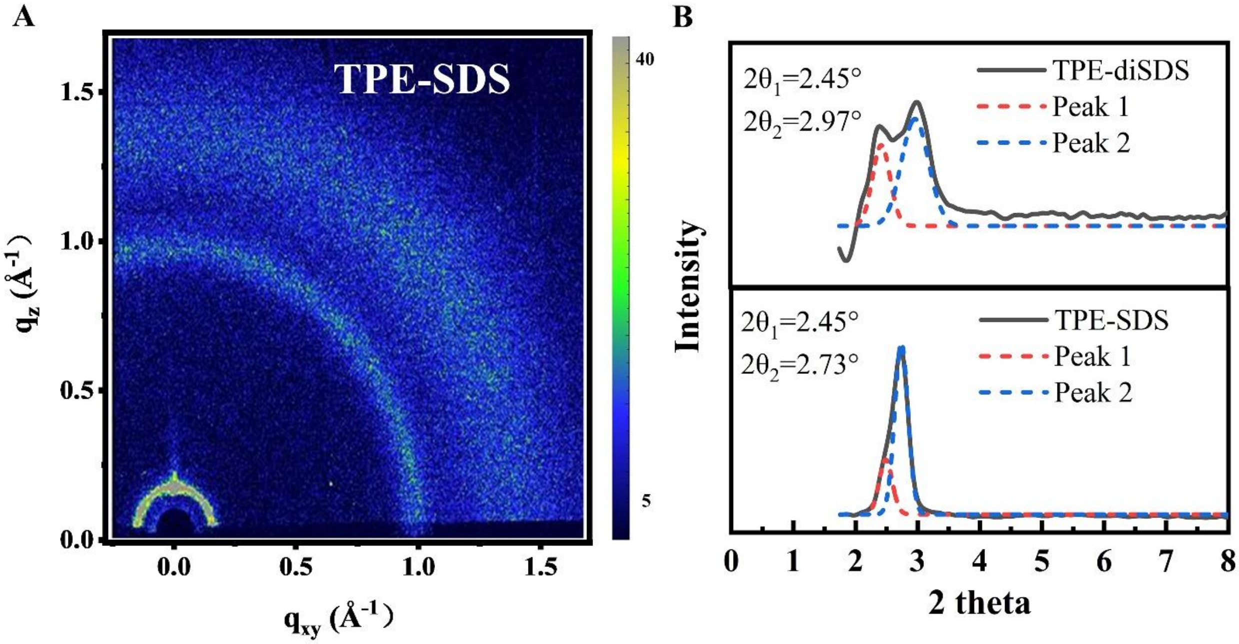

Fig. S15. The supplementary details of GiWAXS. (A) The GIWAXS result of *TPE-SDS* patterns. **(B)** Circle integration and fitting results of the GIWAXS about *TPE-diSDS* and *TPE-SDS*. By comparing the circle integral results of these two molecules, it can be found that the layered structure induced by the solid interface in the edge region is both retained in two model molecules, but the layered structure formed by the gas-liquid-gas interface in *TPE-diSDS* changes to isotropy in *TPE-SDS*.

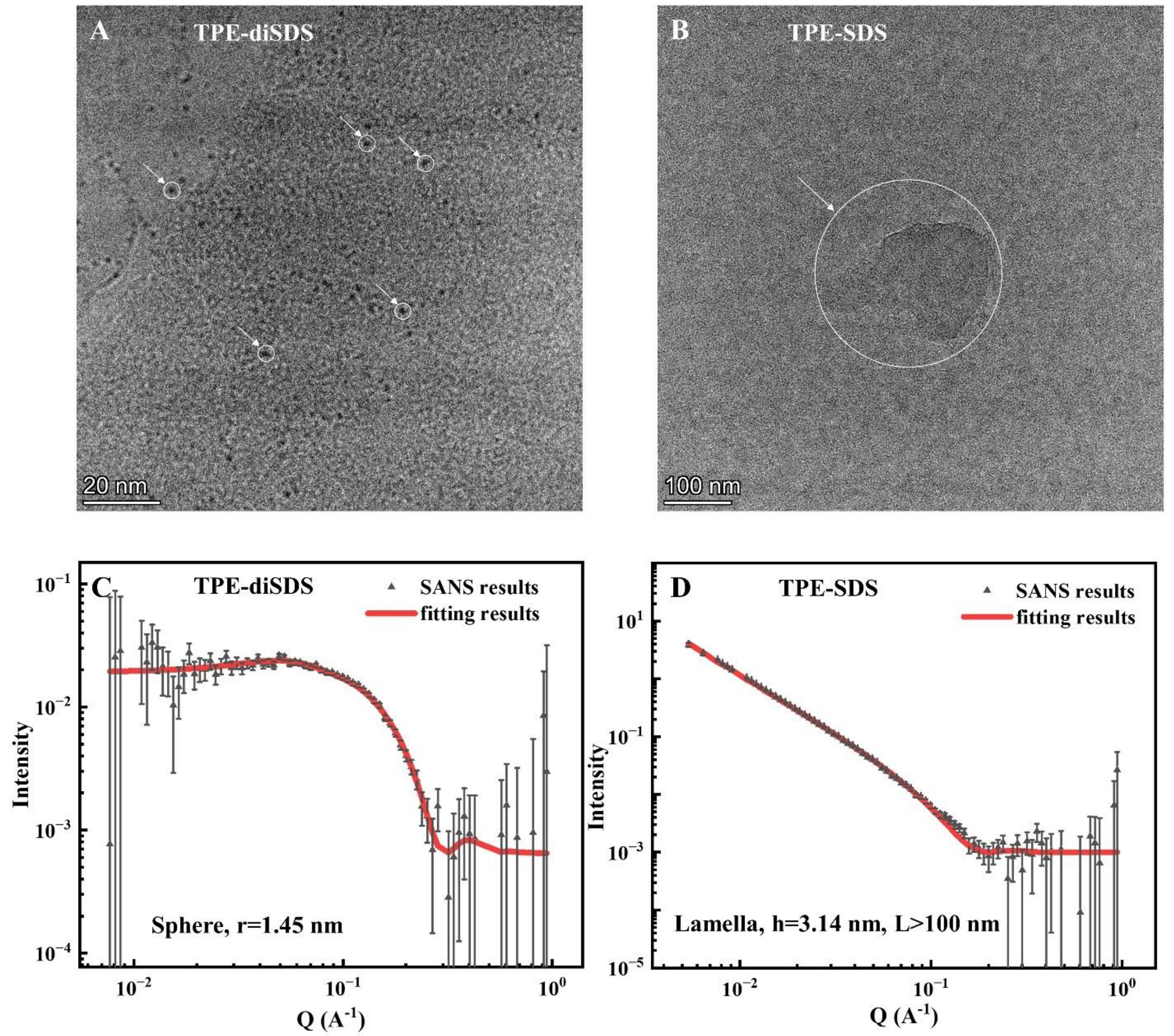

Fig. S16. Supplementary evidence for molecular aggregation in solution (water, pH=2). (A) Cryo-TEM images of *TPE-diSDS* solution which aggregate to small spherical micelle (radius about 1-2 nm). (B) Cryo-TEM images of *TPE-SDS* solution which aggregate to huge lamella micelle (length about 100-200 nm). (C) The small-angle neutron scattering (SANS) results of *TPE-diSDS* solution. The scattering date fits the spherical model well and the radius is approximately 1.45 nm. (D) the SANS results of *TPE-SDS* solution. The scattering data fits the lamella model well. The thickness is 3.14 nm and the length is longer than 100 nm. The concentration of *TPE-diSDS* solution used to finish cryo-TEM and SANS measurement is 2.5 mg/mL, and the concentration of *TPE-SDS* is 2.0 mg/mL.

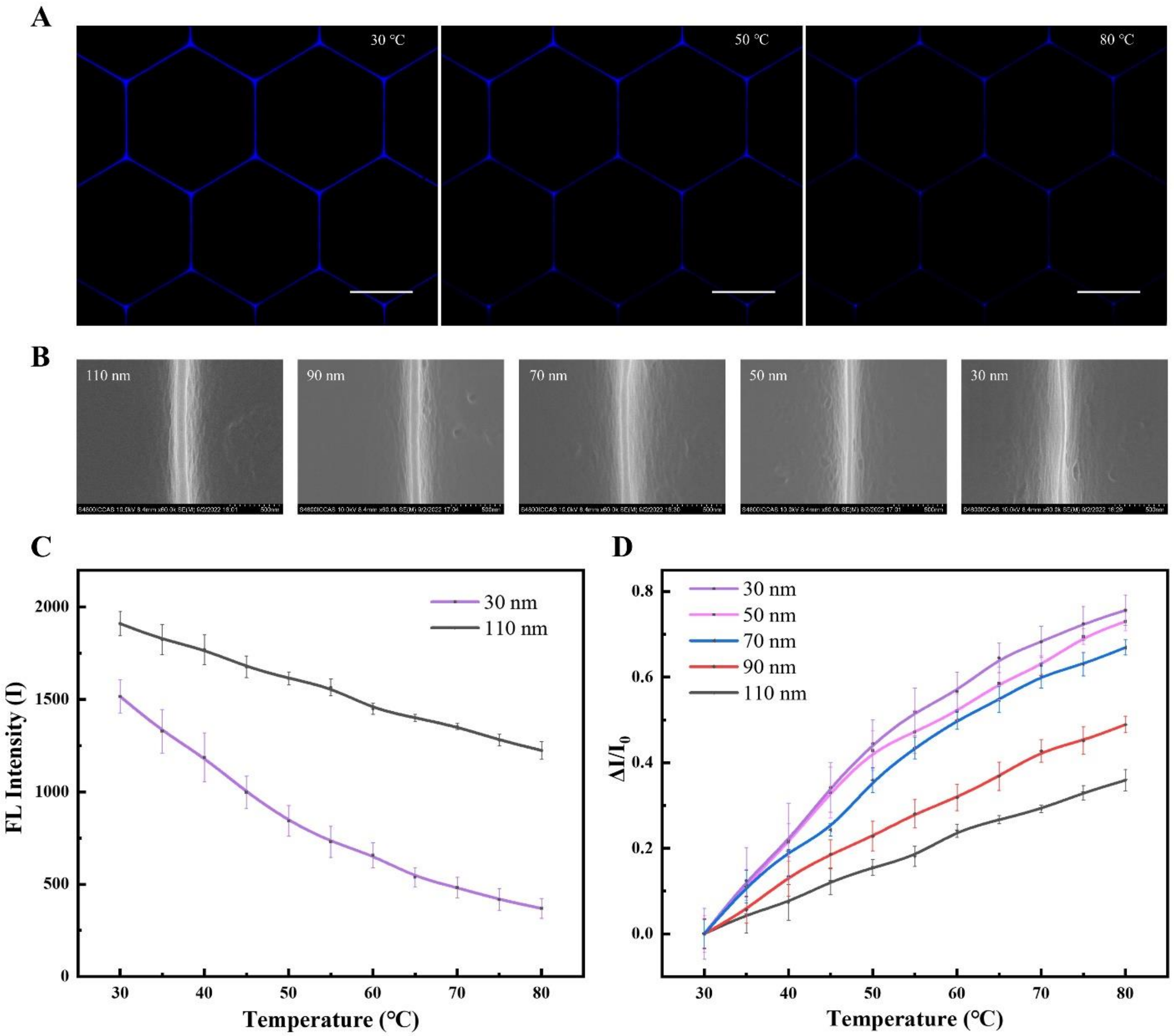

Fig. S17. Sensitive temperature sensor of high-precision molecular patterns based on AIE-property and BTMP method. (A) The fluorescence images of *TPE-diSDS* molecular patterns under various temperature, including 30℃, 50℃ and 80℃. **(B)** The SEM results of molecular patterns with different line width used in temperature-sensor, from 30 nm to 110 nm. **(C)** The fluorescent intensity (I) variations of molecular patterns in response to varying temperature. With the increase of the temperature, the molecular thermal motion is enhanced*(52, 53)*, and the fluorescence insensitivity is weakened. **(D)** The line width dependence of the relative variations of the fluorescence intensity, $\Delta I/I_0$ to different temperature, which shows an upward trend with the improvement of pattern precision. Scale bars in **A** are 50 μm.

REFERENCES AND NOTES

1. W. Cheng, N. Park, M. T. Walter, M. R. Hartman, D. Luo, Nanopatterning self-assembled nanoparticle superlattices by moulding microdroplets. *Nat. Nanotechnol.* **3**, 682–690 (2008).

2. M. S. Onses, C. Song, L. Williamson, E. Sutanto, P. M. Ferreira, A. G. Alleyne, P. F. Nealey, H. Ahn, J. A. Rogers, Hierarchical patterns of three-dimensional block-copolymer films formed by electrohydrodynamic jet printing and self-assembly. *Nat. Nanotechnol.* **8**, 667–675 (2013).

3. T. Gadt, N. S. Ieong, G. Cambridge, M. A. Winnik, I. Manners, Complex and hierarchical micelle architectures from diblock copolymers using living, crystallization-driven polymerizations. *Nat. Mater.* **8**, 144–150 (2009).

4. Y. Qiu, B. Zhang, J. Yang, H. Gao, S. Li, L. Wang, P. Wu, Y. Su, Y. Zhao, J. Feng, L. Jiang, Y. Wu, Wafer-scale integration of stretchable semiconducting polymer microstructures via capillary gradient. *Nat. Commun.* **12**, 7038 (2021).

5. X. Jiang, H. Gao, X. Zhang, J. Pang, Y. Li, K. Li, Y. Wu, S. Li, J. Zhu, Y. Wei, L. Jiang, Highly-sensitive optical organic vapor sensor through polymeric swelling induced variation of fluorescent intensity. *Nat. Commun.* **9**, 3799 (2018).

6. Y.-Q. Zheng, Y. Liu, D. Zhong, S. Nikzad, S. Liu, Z. Yu, D. Liu, H.-C. Wu, C. Zhu, J. Li, Monolithic optical microlithography of high-density elastic circuits. *Science* **373**, 88–94 (2021).

7. H. Tran, K. L. Killops, L. M. Campos, Advancements and challenges of patterning biomolecules with sub-50 nm features. *Soft Matter* **9**, 6578–6586 (2013).

8. R. C. Schmidt, K. E. Healy, Controlling biological interfaces on the nanometer length scale. *J. Biomed. Mater. Res. A* **90**, 1252–1261 (2009).

9. M. Guo, Z. Qu, F. Min, Z. Li, Y. Qiao, Y. Song, Advanced unconventional techniques for sub-100 nm nanopatterning. *InfoMat* **4**, e12323 (2022).

10. S. Hong, J. Zhu, C. A. Mirkin, Multiple ink nanolithography: Toward a multiple-pen nano-plotter. *Science* **286**, 523–525 (1999).

11. G. Liu, S. H. Petrosko, Z. Zheng, C. A. Mirkin, Evolution of dip-pen nanolithography (DPN): From molecular patterning to materials discovery. *Chem. Rev.* **120**, 6009–6047 (2020).

12. R. D. Piner, J. Zhu, F. Xu, S. Hong, C. A. Mirkin, "Dip-Pen" nanolithography. *Science* **283**, 661–663 (1999).

13. L. J. Guo, Nanoimprint lithography: Methods and material requirements. *Adv. Mater.* **19**, 495–513 (2007).

14. A. Espinha, C. Dore, C. Matricardi, M. I. Alonso, A. R. Goni, A. Mihi, Hydroxypropyl cellulose photonic architectures by soft nanoimprinting lithography. *Nat. Photonics* **12**, 343–348 (2018).

15. W. Li, W. Wu, R. S. Williams, Single-digit nanometer nanoimprint templates. *SPIE* 10.1117/2.1201307.004975 (2013).

16. C. Tang, E. M. Lennon, G. H. Fredrickson, E. J. Kramer, C. J. Hawker, Evolution of block copolymer lithography to highly ordered square arrays. *Science* **322**, 429–432 (2008).

17. K. Nickmans, A. Schenning, Directed self-assembly of liquid-crystalline molecular building blocks for sub-5 nm nanopatterning. *Adv. Mater.* **30**, 1703713 (2018).

18. K. Yoshida, L. Tian, K. Miyagi, A. Yamazaki, H. Mamiya, T. Yamamoto, K. Tajima, T. Isono, T. Satoh, Facile and efficient modification of polystyrene-block-poly(methyl methacrylate) for achieving sub-10 nm feature size. *Macromolecules* **51**, 8064–8072 (2018).

19. P. W. Rothemund, Folding DNA to create nanoscale shapes and patterns. *Nature* **440**, 297–302 (2006).

20. A. Aghebat Rafat, S. Sagredo, M. Thalhammer, F. C. Simmel, Barcoded DNA origami structures for multiplexed optimization and enrichment of DNA-based protein-binding cavities. *Nat. Chem.* **12**, 852–859 (2020).

21. J. Zasadzinski, R. Viswanathan, L. Madsen, J. Garnaes, D. Schwartz, Langmuir-Blodgett films. *Science* **263**, 1726–1733 (1994).

22. V. Bergeron, Forces and structure in thin liquid soap films. *J. Phys. Condens. Matter* **11**, R215 (1999).

23. Z. Li, Z. Huang, Q. Yang, M. Su, X. Zhou, H. Li, L. Li, F. Li, Y. Song, Bioinspired anti-moiré random grids via patterning foams. *Adv. Opt. Mater.* **5**, 1700751 (2017).

24. Z. Huang, M. Su, Q. Yang, Z. Li, S. Chen, Y. Li, X. Zhou, F. Li, Y. Song, A general patterning approach by manipulating the evolution of two-dimensional liquid foams. *Nat. Commun.* **8**, 14110 (2017).

25. F. Min, P. Zhou, Z. Huang, Y. Qiao, C. Yu, Z. Qu, X. Shi, Z. Li, L. Jiang, Z. Zhang, X. Yan, Y. Song, A Bubble-assisted approach for patterning nanoscale molecular aggregates. *Angew. Chem. Int. Ed.* **60**, 16547–16553 (2021).

26. J. Marchalot, J. Lambert, I. Cantat, P. Tabeling, M.-C. Jullien, 2D foam coarsening in a microfluidic system. *Europhys. Lett.* **83**, 64006 (2008).

27. C. Ochoa, S. Gao, S. Srivastava, V. Sharma, Foam film stratification studies probe intermicellar interactions. *Proc. Natl. Acad. Sci. U.S.A.* **118**, 2024805118 (2021).

28. Z. Qu, J. Qin, F. Min, Z. Huang, Z. Li, Z. Cai, Y. Qiao, Y. Song, Micro-bubble manipulation and its application in functional material patterning. *Sci. Sin. Chim.* **52**, 209–221 (2022).

29. M. J. Sun, Y. Liu, W. Zeng, Y. S. Zhao, Y. W. Zhong, J. Yao, Photoluminescent anisotropy amplification in polymorphic organic nanocrystals by light-harvesting energy transfer. *J. Am. Chem. Soc.* **141**, 6157–6161 (2019).

30. S. D. Hiremath, R. U. Gawas, S. C. Mascarenhas, A. Ganguly, M. Banerjee, A. Chatterjee, A water-soluble AIE-gen for organic-solvent-free detection and wash-free imaging of Al^{3+} ions and subsequent sensing of F^{-} ions and DNA tracking. *New J. Chem.* **43**, 5219–5227 (2019).

31. W. Guan, W. Zhou, C. Lu, B. Z. Tang, Synthesis and design of aggregation-induced emission surfactants: Direct observation of micelle transitions and microemulsion droplets. *Angew. Chem. Int. Ed.* **54**, 15160–15164 (2015).

32. S. I. Kemal, C. A. Uribe Ortiz, V. Sharma, Surface forces and stratification in foam films formed with bile salts. *Mol. Syst. Des. Eng.* **6**, 520–533 (2021).

33. M. J. Frisch, G. W. Trucks, H. B. Schlegel, G. E. Scuseria, M. A. Robb, J. R. Cheeseman, G. Scalmani, V. Barone, G. A. Petersson, H. Nakatsuji, X. Li, M. Caricato, A. V. Marenich, J. Bloino, B. G. Janesko, R. Gomperts, B. Mennucci, H. P. Hratchian, J. V. Ortiz, A. F. Izmaylov, J. L. Sonnenberg, Williams, F. Ding, F. Lipparini, F. Egidi, J. Goings, B. Peng, A. Petrone, T. Henderson, D. Ranasinghe, V. G. Zakrzewski, J. Gao, N. Rega, G. Zheng, W. Liang, M. Hada, M. Ehara, K. Toyota, R. Fukuda, J. Hasegawa, M. Ishida, T. Nakajima, Y. Honda, O. Kitao, H. Nakai, T. Vreven, K. Throssell, J. A. Montgomery Jr., J. E. Peralta, F. Ogliaro, M. J. Bearpark, J. J. Heyd, E. N. Brothers, K. N. Kudin, V. N. Staroverov, T. A. Keith, R. Kobayashi, J. Normand, K. Raghavachari, A. P. Rendell, J. C. Burant, S. S. Iyengar, J. Tomasi, M. Cossi, J. M. Millam, M. Klene, C. Adamo, R. Cammi, J. W. Ochterski, R. L. Martin, K. Morokuma, O. Farkas, J. B. Foresman, D. J. Fox, Gaussian 09 Revision D.01 (Gaussian Inc., 2016).

34. T. Lu, F. Chen, Multiwfn: A multifunctional wavefunction analyzer. *J. Comput. Chem.* **33**, 580–592 (2012).

35. W. Humphrey, A. Dalke, K. Schulten, VMD: Visual molecular dynamics. *J. Mol. Graph.* **14**, 33–38 (1996).

36. D. Van Der Spoel, E. Lindahl, B. Hess, G. Groenhof, A. E. Mark, H. J. Berendsen, GROMACS: Fast, flexible, and free. *J. Comput. Chem.* **26**, 1701–1718 (2005).

37. B. R. Brooks, R. E. Bruccoleri, B. D. Olafson, D. J. States, S. Swaminathan, M. Karplus, CHARMM: A program for macromolecular energy, minimization, and dynamics calculations. *J. Comput. Chem.* **4**, 187–217 (1983).

38. B. Hess, H. Bekker, H. J. Berendsen, J. G. Fraaije, LINCS: A linear constraint solver for molecular simulations. *J. Comput. Chem.* **18**, 1463–1472 (1997).

39. T. Darden, D. York, L. Pedersen, Particle mesh Ewald: An $N \cdot \log(N)$ method for Ewald sums in large systems. *J. Chem. Phys.* **98**, 10089–10092 (1993).

40. W. G. Hoover, Canonical dynamics: Equilibrium phase-space distributions. *Phys. Rev. A. Gen. Phys.* **31**, 1695–1697 (1985).

41. O. Cherniavskaya, A. Adzic, C. Knutson, B. J. Gross, L. Zang, R. Liu, D. M. Adams, Edge transfer lithography of molecular and nanoparticle materials. *Langmuir* **18**, 7029–7034 (2002).

42. T. W. Odom, V. R. Thalladi, J. C. Love, G. M. Whitesides, Generation of 30–50 nm structures using easily fabricated, composite PDMS masks. *J. Am. Chem. Soc.* **124**, 12112–12113 (2002).

43. J. Aizenberg, A. J. Black, G. M. Whitesides, Controlling local disorder in self-assembled monolayers by patterning the topography of their metallic supports. *Nature* **394**, 868–871 (1998).

44. S. Dallorto, D. Staaks, A. Schwartzberg, X. Yang, K. Y. Lee, I. W. Rangelow, S. Cabrini, D. L. Olynick, Atomic layer deposition for spacer defined double patterning of sub-10 nm titanium dioxide features. *Nanotechnology* **29**, 405302 (2018).

45. B. Su, S. Wang, J. Ma, Y. Wu, X. Chen, Y. Song, L. Jiang, Elaborate positioning of nanowire arrays contributed by highly adhesive superhydrophobic pillar-structured substrates. *Adv. Mater.* **24**, 559–564 (2012).

46. B. Su, S. Wang, Y. Wu, X. Chen, Y. Song, L. Jiang, Small molecular nanowire arrays assisted by superhydrophobic pillar-structured surfaces with high adhesion. *Adv. Mater.* **24**, 2780–2785 (2012).

47. B. Su, C. Zhang, S. Chen, X. Zhang, L. Chen, Y. Wu, Y. Nie, X. Kan, Y. Song, L. Jiang, A general strategy for assembling nanoparticles in one dimension. *Adv. Mater.* **26**, 2501–2507 (2014).

48. A. Pandey, S. Tzadka, D. Yehuda, M. Schvartzman, Soft thermal nanoimprint with a 10 nm feature size. *Soft Matter* **15**, 2897–2904 (2019).

49. J. Jiang, A. G. Jacobs, B. Wenning, C. Liedel, M. O. Thompson, C. K. Ober, Ultrafast self-assembly of sub-10 nm block copolymer nanostructures by solvent-free high-temperature laser annealing. *ACS Appl. Mater. Interfaces* **9**, 31317–31324 (2017).

50. R. Chikkaraddy, V. A. Turek, N. Kongsuwan, F. Benz, C. Carnegie, T. van de Goor, B. de Nijs, A. Demetriadou, O. Hess, U. F. Keyser, J. J. Baumberg, Mapping nanoscale hotspots with single-molecule emitters assembled into plasmonic nanocavities using DNA origami. *Nano Lett.* **18**, 405–411 (2018).

51. F. N. Gur, C. P. T. McPolin, S. Raza, M. Mayer, D. J. Roth, A. M. Steiner, M. Loffler, A. Fery, M. L. Brongersma, A. V. Zayats, T. A. F. Konig, T. L. Schmidt, DNA-Assembled plasmonic waveguides for nanoscale light propagation to a fluorescent nanodiamond. *Nano Lett.* **18**, 7323–7329 (2018).

52. P. Yao, W. Qiao, Y. Wang, H. Peng, X. Xie, Z. Li, Deep-red emissive squaraine-AIEgen in elastomer enabling high contrast and fast thermoresponse for anti-counterfeiting and temperature sensing. *Chemistry* **28**, e202200725 (2022).

53. N. B. Shustova, T.-C. Ong, A. F. Cozzolino, V. K. Michaelis, R. G. Griffin, M. Dincă, Phenyl ring dynamics in a tetraphenylethylene-bridged metal–organic framework: Implications for the mechanism of aggregation-induced emission. *J. Am. Chem. Soc.* **134**, 15061–15070 (2012)
